# IND-enabling preclinical studies of [^11^C]COU, a trapped metabolite PET radiotracer for monoamine oxidase-B

**DOI:** 10.1007/s00044-025-03496-0

**Published:** 2025-11-05

**Authors:** Madison S. Frazier, Tanpreet Kaur, Jenelle Stauff, Wade P. Winton, Bradford D. Henderson, Alexandra S. Dumond, Xia Shao, David M. Raffel, Kirk A. Frey, Michael R. Kilbourn, Allen F. Brooks, Peter J. H. Scott

**Affiliations:** 1 Department of Medicinal Chemistry, College of Pharmacy, University of Michigan, Ann Arbor, MI 48109 USA; 2 Department of Radiology, University of Michigan, Ann Arbor, MI 48109 USA; 3 Department of Pharmacology, University of Michigan, Ann Arbor, MI 48109 USA

**Keywords:** PET imaging, Preclinical study, MAO-B, Synthesis validation, Dosimetry, Pharmacology/Toxicology

## Abstract

[^11^C]COU is a trapped metabolite radiotracer for in vivo analysis of Monoamine Oxidase B activity using positron emission tomography (PET) imaging. [^11^C]COU has the potential to quantify astrocytosis in the early stages of Alzheimer’s disease, providing an earlier marker of disease than currently available for staging disease progression. Prior preclinical studies have demonstrated the efficacy of this radiotracer in preclinical imaging studies, warranting the translation for clinical evaluation. In this paper, we describe results of the requisite preclinical studies required to obtain approval for translation of [^11^C]COU into first-in-human studies. Development and validation of a production method that conforms to the quality requirements described in the US Pharmacopeia was accomplished, along with preclinical rodent studies to determine human radiation dose estimates and a single acute dose pharmacology and toxicology study to establish that an injected mass dose 100-fold higher than the proposed PET imaging dose was below the no-observed-adverse-effect level (NOAEL). The production method was validated in triplicate, yielding [^11^C]COU in sufficient radiochemical yield (9.3 ± 0.008%), radiochemical purity (99.2 ± 0.002%) and molar activity (165 ± 65 TBq/mmol) for routine clinical use, and providing a product that was sterile and met (or exceeded) all quality control requirements for human use. Dosimetric analysis determined that the effective human dose of [^11^C]COU is 0.005 mSv/MBq, also acceptable for clinical use. Lastly, no observable adverse effects were noted at 86 μg/kg in rodent toxicology studies (100x the proposed human dose). From these results we received approval to advance [^11^C]COU into clinical studies.

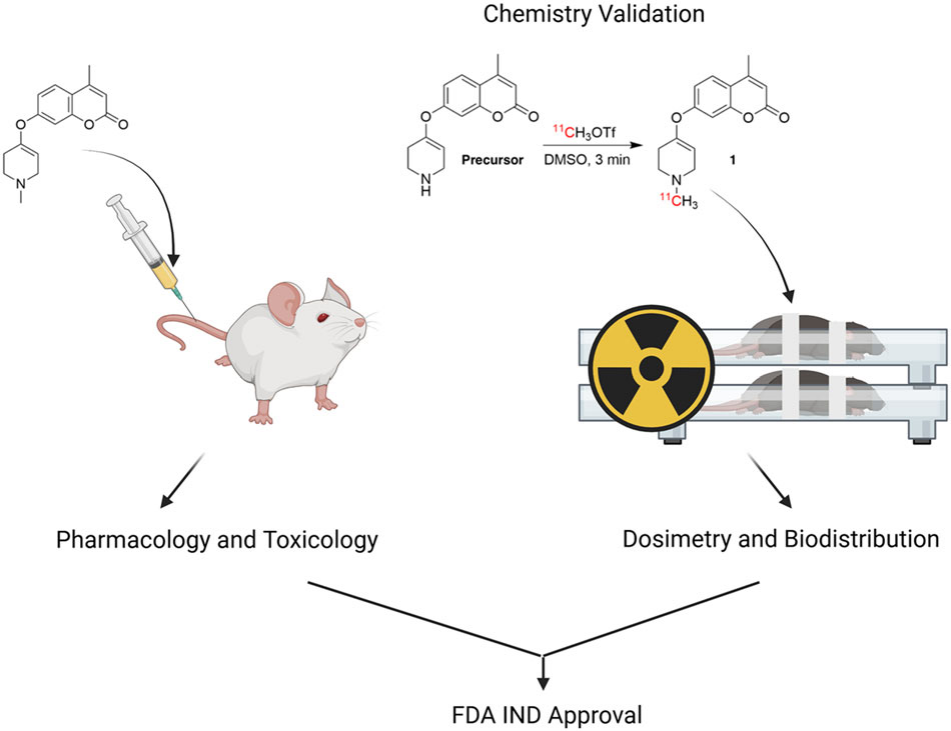

## Introduction

As the global population ages, the incidence of Alzheimer’s Disease and related dementias (ADRD) is expected to increase, thus the need for earlier clinical diagnosis is essential [1]. The current standard of care involves measuring amyloid β (Aβ) plaques and tau neurofibrillary tangles (NFTs) either through blood tests or Positron Emission Tomography (PET) imaging [2]. Treatments to alleviate Aβ burden, lecanemab and donanemab, are effective at removing plaques from the brain and have recently received FDA approval for treatment of AD. However, when Aβ and tau accumulation become measurable, neuronal impairment and loss has already begun and thus alleviation of symptoms with these treatments is somewhat limited, yet disease progression has been slowed with the use of these new drugs [3, 4].

The process of plaque deposition and NFT formation have been shown to begin years before symptoms arise in ADRD [5–9]. Due to this disconnect in disease onset and diagnosis, many researchers have begun to look for other biomarkers that can be detected earlier in AD progression. Currently the earliest detectable change in patients who will go on to develop AD is neuroinflammation [5, 6]. There are two cells that play a role in this process, microglia and astrocytes [10]. Normally these cells maintain the homeostasis of the neurological system by keeping ions in balance, metabolizing neurotransmitters, and maintaining the blood-brain barrier (BBB) [11]. However, as Aβ fibrils begin to build up in the brain, these cells become hyperactivated in a process called gliosis or astrocytosis [12]. As neuroinflammation emerges as an early biomarker of ADRD, and modulating immune responses is considered as a potential therapeutic strategy, there is growing interest in quantifying neuroinflammation in ADRD via PET imaging [10, 13, 14]. Several approaches have used PET to detect activated microglia. For example, the translocator protein 18 kDa (TSPO), expressed on activated microglia, has been widely used for the quantification of neuroinflammation in AD [15]. Evidence suggests microglial activation precedes regional tau and neurodegeneration in AD and, reflecting this, higher uptake of TSPO radiotracers such as [^11^C]PBR28 has been associated with higher Aβ and tau burden, as well as lower MMSE score [16]. PET imaging of cyclooxygenase 1 (COX1) [17–19] and P2X ligand-gated ion channel type 7 (P2X7R) [20–22] are also being evaluated since both targets have been detected at higher levels in post-mortem brain tissue from AD subjects [23], with promising imaging agents for both advancing into human studies. These targets enable detection of the change in microglial expression or general neuroinflammation.

An alternative approach to imaging neuroinflammation is detection of astrocytosis [24]. There are a number of ways imaging researchers are trying to accomplish this, including targeting imidazoline_2_ binding sites (I_2_BS) expressed in the outer mitochondrial membrane of astrocytes, as well as evaluating whether TSPO comes mainly from astrocytes later in the inflammatory cascade [25]. However, the target that has seen the most development for PET imaging in the context of reactive astrocytes is monoamine oxidase B (MAO-B) [26]. MAO-B is a metabolic enzyme that is found on the outer mitochondrial membrane of astrocytes. Its main function is to metabolize endogenous monoamine neurotransmitters such as dopamine and norepinephrine [27]. MAO-B is a particularly attractive imaging target as it exists predominantly in astrocytes, with only a small quantity expressed in serotonergic neurons. This high specificity is beneficial for PET imaging as it gives clear imaging results and enables straightforward data analysis [28]. Since the pioneering work of Fowler and others in the 1980s [29], several radiotracers have been evaluated for clinical PET imaging of MAO-B, including: [^11^C/^18^F]deprenyl as well as their deuterated derivatives [6, 30–34], [^11^C]SL2511.88 [35–38], [^18^F]THK5351 [39], and [^18^F]SMBT [40, 41]. A number of these tracers have been used for imaging ADRD, with demonstrated specificity for MAO-B over MAO-A, and discerning differences in healthy control subjects, subjects with mild cognitive impairment (MCI), and later staged AD subjects [30, 35, 40]. Studies with [^11^C]deprenyl have also illuminated longitudinal changes in astrocytosis, Aβ accumulation, and glucose hypometabolism in subjects with autosomal dominant AD [9], demonstrating that changes in all these parameters can be detected prior to changes in clinical cognition. However, a limitation of all the current radiotracers for MAO-B is that they are based upon inhibitors of the enzyme. As such, while they enable quantitation of the amount and location of MAO-B, they provide no information on its metabolic activity or functional state.

The ability to quantify MAO-B activity using PET would allow for further investigation into the metabolic state of the astrocytes in AD brains. This is of interest as astrocytes can exist in various different morphologies, which serve different roles in the brain [11]. For instance, neuroinflammation can be beneficial, such as when it promotes scar tissue formation around damaged areas of the brain following stoke or TBI [42]. Conversely, when it becomes chronic, neuroinflammation is a prolonged and uncontrolled inflammatory response that can become self-propagating and harmful, leading to neuron damage, loss of function, and neurodegeneration, as seen in ADRD [43]. Being able to discern the type of neuroinflammation that is occurring would greatly improve our knowledge of the role these cells play, and this is crucial to understand in the case of leveraging anti-inflammatory agents as a potential ADRD treatment strategy.

**Fig. 1:**
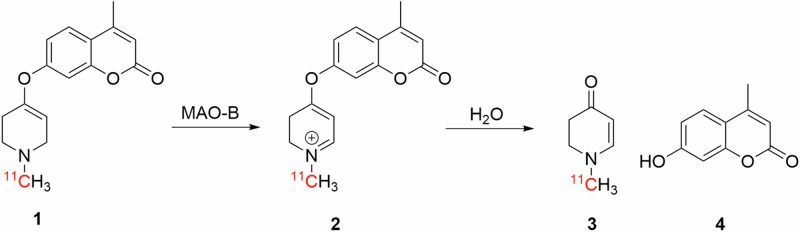
Proposed mechanism of trapping for [^11^C]COU

We hypothesized a substrate-based radiotracer that, following oxidation by MAO-B, results in a labeled trapped metabolite would enable quanitifcation of MAO-B activity, akin to [^18^F]fluorodeoxyglucose (FDG) or N-[^11^C]methylpiperidin-4-yl propionate (PMP) for hexokinase-2 and acetylcholinesterase, respectively [44–47]. In marked constrast to inhibitor-based imaging agents which provide information on enzyme expression levels, the rate of trapping of a substrate-based tracer reflects enzyme activity. To this end, our group first published in vivo imaging of MAO-B with the radiotracer 4-methyl-7-[(1-[^11^C]methyl-1,2,3,6-tetrahydropyridin-4-yl)-oxy]-2Hchromen-2-one ([^11^C]COU, **1**) in 2015 (Fig. 1) [48]. This radiotracer was inspired by the evaluation of MAO-B metabolism using analogues of the neurotoxin 1-methyl-4-phenyl-1,2,3,6-tetrahydropyridine (MPTP) pioneered by the Castagnoli lab [49, 50]. Castagnoli showed that adding an ether linkage between the phenyl group and the tetrahydropyridine (THP) in the MPTP structure allowed the molecule to be cleaved by the enzyme resulting in two non-toxic metabolites, rather than generating MPP^+^ which becomes trapped in dopaminergic neurons leading to neuronal cell death and Parkinsonian symptoms [50–52].

We have previously shown that [^11^C]COU penetrates the CNS in rodents and nonhuman primates, and that the PET signal in the brain can be reduced by pretreatment with MAO-B inhibitors such as deprenyl [48, 51, 53]. [^11^C]COU is first oxidized by MAO-B to give intermediate **2**, which is subsequently hydrolyzed to generate 1-methyl-2,3-dihydropyridin-4(1H)-one (**3**) and coumarin (**4**) (Fig. 1). The small polar metabolite **3** is trapped in the brain, suggesting that kinetic modeling can be used to quantify MAO-B activity in the future [42–45]. In preliminary safety studies, [^11^C]COU was also shown to be non-toxic by using [^11^C]DTBZ PET to show that dopaminergic neurons, which are damaged by neurotoxic MPTP, are unaffected by [^11^C]COU [48, 53, 54]. Taken together, the promising preclinical imaging studies and favorable safety profile motivated us to translate [^11^C]COU into the clinic. In order to translate [^11^C]COU into first-in-human studies, it is required to validate the radiosynthesis according to GMP regulations, and demonstrate in vivo safety through preclinical estimates of radiation absorbed dose (dosimetry) and single species pharmacology/toxicology studies. Completion of these IND-enabling studies is the focus of this report.

## Results and discussion

### Radiochemistry validation

COU precursor and reference standard ([^12^C]**2**) standard were synthesized at gram scale using reported procedures (see Supporting Information for details). Both the standard and precursor lots were analyzed by NMR and analytical HPLC annually for purity and repurified when necessary. These compounds were then aliquoted into 1 mg lots and stored in a −20 °C freezer with desiccant until use. Both the reference standard and precursor are stable when stored in this fashion, and annual recertification has revealed no appreciable decomposition over at least 5 years.

**Fig. 2:**
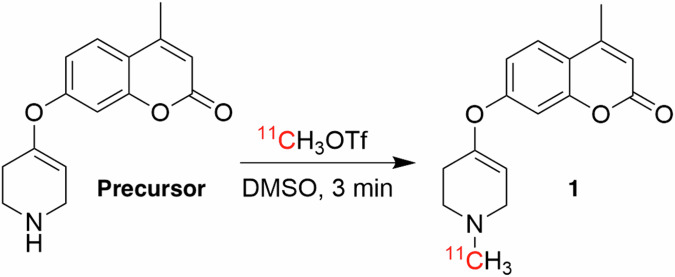
Synthesis of [^11^C]COU (**1**) via [^11^C]methylation of free amine COU precursor. Synthesis performed on GE TracerLab FX_C-Pro_ platform

Next, three process verification runs preparing [^11^C]COU were completed in succession over the span of two days. These verification runs were undertaken by ^11^C-methylation of the des-methyl precursor according to our published procedures (Fig. 2). Briefly, in a TRACERLab FX_C-Pro_ synthesis module, the precursor is methylated with [^11^C]MeOTf, purified by semi-preparative HPLC and reconstituted into ethanolic saline using a C18 SPE cartridge. The product is then terminally sterilized through a 0.22 µm filter into a sterile dose vial, and a sample of the final product submitted for QC testing. All three syntheses were successful, with an average radiochemical yield of 9.3 ± 0.008%, radiochemical purity of 99.2 ± 0.002% and molar activity of 165.4 ± 64.5 GBq/μmol (n = 3). In each case, the [^11^C]COU product met or exceeded all QC release criteria (Table 1), confirming suitability of the product for clinical use and validating the radiosynthesis method for routine production of [^11^C]COU in future clinical trials.

**Table 1:** The release criteria for [^11^C]COU as established by USP <823> with the results from the three validation runs and pass (met or exceeded the release criteria)/ fail (did not meet the release criteria) qualification

	Release Criteria	Dose 1	Dose 2	Dose 3	Pass/Fail
Batch Size	N/A	2.98 GBq	3.61 GBq	3.7 GBq	Pass
Radiochemical Yield (decay corrected)	N/A	8.26%	9.37%	10.27%	Pass
Radiochemical Purity	≥ 90%	99.50%	98.90%	99.18%	Pass
Total COU Concentration	≤ 1.6 μg/mL	1.0168 μg/mL	0.53 μg/mL	0.43 μg/mL	Pass
Total Impurity Concentration	≤ 1 μg/mL	N/D	N/D	0.0739 μg/mL	Pass
Radioactive Strength	≥ 1.85 GBq/10 mL	3 GBq/10mL	3.6 GBq/10mL	3.7 GBq/10 mL	Pass
Specific Activity	≥ 500 Ci/mmol	2132 Ci/mmol	4961 Ci/mmol	6321 Ci/mmol	Pass
pH	4.5–7.5	5	5	5	Pass
Visual Inspection	Clear, colorless, no precipitate	Pass	Pass	Pass	Pass
Radiochemical Identity	RRT: 0.9–1.1	1.02	1.019	1.02	Pass
Radionuclide Identity	18.4–22.4 min	20.08 min	20.52 min	20.39 min	Pass
Filter Membrane Integrity	≥ 44 psi	48 psi	47.5 psi	47 psi	Pass
Bacterial Endotoxin Test	< 17.5 EU/mL	< 2.00 EU/mL	< 2.00 EU/mL	< 2.00 EU/mL	Pass
Residual Solvent Analysis	Acetone: ≤ 5000 μg/mL	Acetone: 9 μg/mL	Acetone: 8 μg/mL	Acetone: 9 μg/mL	Pass
	MeCN: ≤ 410 μg/mL	MeCN: 5 μg/mL	MeCN: 16 μg/mL	MeCN: 16 μg/mL	Pass
	DMSO: ≤ 5000 μg/mL	DMSO: 15 μg/mL	DMSO: 42 μg/mL	DMSO: N/D μg/mL	Pass
	Total: ≤ 10,000 μg/mL	Total: 29 μg/mL	Total: 66 μg/mL	Total: 25 μg/mL	Pass
Sterility Test	Complies with USP<71>	Pass	Pass	Pass	Pass

### Dosimetry and biodistribution

**Fig. 3:**
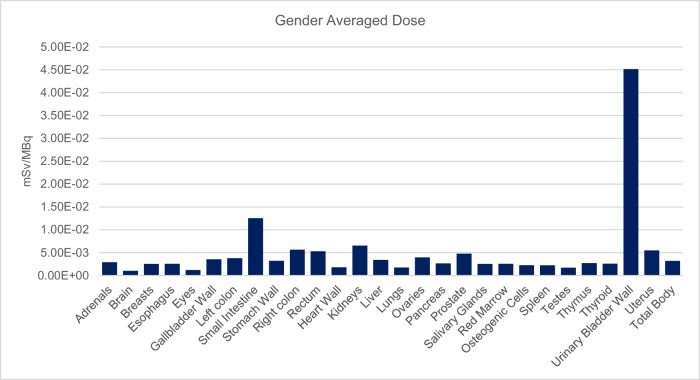
Calculated rodent dosimetry for each major tissue as well as whole body dose

To determine the acceptable human dose of radioactivity from [^11^C]COU, four Sprague Dawley rats (2 male, 2 female) for each of four time points were anesthetized with isoflurane and injected via the femoral vein with 5.55–18.5 MBq of [^11^C]COU [55]. The rats were allowed to recover from the anesthesia and roam freely. These rats were then sacrificed via decapitation at 10-, 20-, 60- and 120-minutes post-injection. Tissues of interest were collected and homogenized. Homogenates were counted using an auto-gamma counter, and the data was used to determine in vivo biodistribution (Supplementary Table 1). The biodistribution data in turn was used to calculate rodent dosimetry (i.e. effective dose) from [^11^C]COU for organs and total body (Fig. 3).

From the calculated biodistribution, medical internal radiation dose (MIRD) formalism using the OLINDA/EXM software was calculated to determine the dosimetry (Table 2) [56]. Consistent with the short half-life of carbon-11 (20 min), dosimetry burden is estimated to be low (Table 2). Brain uptake (and trapping) was consistent with our prior reports (ca. 0.16% injected dose per gram (ID/g) 10 min post-injection) [48], and organs such as small intestine, liver and kidneys have higher uptake due to excretion of [^11^C]COU (e.g. kidneys had ca. 1.5% ID/g 10 min post-injection). Reflecting this, organs with metabolic or excretory activity are estimated to receive the highest doses of radiation. Since [^11^C]COU (and its polar labeled-metabolite) clear mainly via urinary excretion, urinary bladder wall was estimated to receive the largest dose (0.0452 mSv/MBq). Applying the guidelines of radioactive drug research committees (RDRCs) operating in the United States, the dose limit for adults is defined as follows: 30 mSv per single injection for the whole body, lens of the eyes, red marrow, and gonads; 50 mSv per year for the whole body, lens of the eyes, red marrow, and gonads; 50 mSv per single injection for all other organs; and 150 mSv per year for all other organs (FDA 21 CFR 361.1). Notably, each organ as well as the whole-body calculation for radiation exposure fell below safety guidelines. The estimated dosimetry for [^11^C]COU thus allows administration of up to 2 × 666 MBq doses of [^11^C]COU in future clinical studies with an effective dose of 3.06 mSv, which falls within the acceptable limits established by the RDRC.

**Table 2:** Estimated human equivalent dosimetry for each major tissue as well as whole body dose.

Target Organ	Gender Averaged Dose (mSv/MBq)	Target Organ	Gender Averaged Dose (mSv/MBq)
Adrenals	2.91E–03	Lungs	1.77E–03
Brain	1.05E–03	Ovaries	3.97E–03
Breasts	2.56E–03	Pancreas	2.65E–03
Esophagus	2.58E–03	Prostate	4.78E–03
Eyes	1.21E–03	Salivary Glands	2.56E–03
Gallbladder Wall	3.55E–03	Red Marrow	2.58E–03
Left colon	3.79E–03	Osteogenic Cells	2.28E–03
Small Intestine	1.26E–02	Spleen	2.24E–03
Stomach Wall	3.23E–03	Testes	1.71E–03
Right colon	5.65E–03	Thymus	2.71E–03
Rectum	5.31E–03	Thyroid	2.60E–03
Heart Wall	1.81E–03	Urinary Bladder Wall	4.52E–02
Kidneys	6.55E–03	Uterus	5.50E–03
Liver	3.43E–03		
**Total Body**		**3.20E–03**	
**Effective Dose:**		**4.60E–03**	

Bold values are the total body dose for rats and the effective dose estimate.

### Pharmacology and toxicology studies

A single-species pharm-tox study to assess preclinical safety of COU was conducted in Sprague-Dawley rats. Rodents were given either a single injection of 86 μg/kg of COU (100x the anticipated dose for future clinical PET imaging studies) (18 male, 18 female) or 5% ethanol in saline vehicle (4 male, 4 female) and sacrificed either 2–4- or 15-days post-injection to look for acute or sub-acute effects, respectively.

Parameters for evaluation included clinical observations, body weights, food intake, and physical examinations, including ophthalmology. Additionally, organ weights were recorded and gross (macroscopic) pathological examinations were performed on all study animals at necropsy. Hematology and clinical chemistry evaluations were performed on blood samples collected from all study animals just prior to necropsy. Histopathological (microscopic) examinations were performed on the tissues collected at necropsy.

All measurements taken during this study were within normal limits and no drug-related adverse reactions were reported and no drug-related clinical findings. Body weight stayed steady through the study with a mean weight of 211 g at baseline and 285 g on day 15 for males and 214 g at baseline and 233 g on day 15 for females. This increase follows normal weight gain for rats [57]. Food consumption also stayed consistent with an average of 21.9 ± 1.8 g/day for males and 17.2 ± 1.6 g/day for females. All organs measured weighed similar from day 2–4 to day 15. All blood chemistry measurements and blood cell counts were not significantly changed between days 2–4 and 15. In the animals sacrificed at days 2–4, a low grade intermyelinic edema was observed. In a supplemental study, this was investigated further and since both vehicle- and COU-treated animals had similar brain lesions, it was determined that these lesions were not directly COU related (see Supplementary Information). Instead, the vacuolation in brain appears to be vehicle-related or to be due to specimen processing. White matter vacuolation is a very common postmortem artifact associated with fixation if there is excessive exposure to alcohol (https://ntp.niehs.nih.gov/nnl/nervous/brain/index.htm). The fact that formulated COU and vehicle both contained 5% ethanol USP (v/v), offers a possible explanation for this finding.

From this study, COU was determined to have a safety profile appropriate for clinical translation, with a maximum tolerated dose of COU ≥ 86 μg/kg given as a single intravenous injection. This corresponds to the human equivalent dose being ≥14 μg/kg. From the toxicology analysis, the effective dose for use in future clinical studies with [^11^C]COU was set to ≤0.14 μg/kg, 100 x below the NOAEL limit estimated from the rodent pharmacology – toxicology studies.

## Materials and methods

### Synthesis validation

#### General considerations

Detailed experimental protocol, including mass spectrometry, nuclear magnetic resonance (NMR) spectra, semi-preparative and analytical high-performance liquid chromatography (HPLC) traces, are provided in the Supporting Information accompanying this article.

#### Large scale synthesis of cou precursor and standard

To aide in clinical translation, the synthesis of COU precursor and standard were modified to gram scale. This was done with optimization of the synthesis reported in Brooks, et al. [53]. We synthesized 421 mg of precursor at 97% purity and 378 mg of standard using the procedure reported by Brooks, et al. [48] (see see Supplementary Information for more information). Both the standard and precursor lots were analyzed by NMR and analytical HPLC annually for purity and repurified when necessary. These compounds were then aliquoted into 1 mg lots and stored in a −20 °C freezer until use.

*Radiosynthesis of [*^*11*^*C]COU:* Production of ^11^C-labeled radiotracers conducted using General Electric (GE) TRACERLab FX_C-Pro_ automated radiochemistry synthesis module. [^11^C]Carbon dioxide ([^11^C]CO_2_) was produced via ^14^N(p,α)^11^C nuclear reaction using a GE PET Trace cyclotron (60 μA beam for 30 min) and converted by standard procedures into ^11^C-labeled methyl triflate ([^11^C]CH_3_OTf) from ^11^C-labeled methyl iodine ([^11^C]CH_3_I) (25.9 GBq for 30-minute beam). COU precursor (1 mg) was dissolved in DMSO (100 μL) and added to TRACERLab reactor. [^11^C]CH_3_OTf was bubbled through the precursor solution for 3 minutes at room temperature at a rate of 15 mL/min. HPLC buffer (1 mL, 50% MeCN:H_2_O, 50 mM NH_4_HCO_3_, pH 10) was then added to dilute and quench the reaction mixture. The crude reaction mixture was purified using semi-preparative HPLC (column: Gemini 250 × 10 mm−5 μ Luna C18, flow rate: 4 mL/min; 50% MeCN:H_2_O, 50 mM NH_4_HCO_3_, pH 10). The peak corresponding to [^11^C]COU was collected and reformulated by diluting in 50 mL of water and trapping the product on a C18 1cc solid phase extraction (SPE) cartridge. Following trapping, the SPE cartridge was rinsed with water (5 mL) to remove residual MeCN. [^11^C]COU was then eluted with ethanol USP (0.5 mL) and diluted with saline USP (9.5 mL) to produce a formulated dose (5% ethanol in saline) that was then filtered through a 0.22 µm sterile filter into a sterile vial. Following terminal sterilization, a sample (0.5 mL) is removed from the batch and submitted for quality control (QC) testing as described below. The process is accomplished in an overall synthesis times of 30 min from end-of-bombardment (EOB).

### Quality control

All quality control analyses were performed according to requirements described in US Pharmacopeia Chapter <823> and United States Code 21 USC 321. All release criteria are shown with acceptable thresholds along with a pass/fail qualification in Table 1.

#### Analytical HPLC

Both chemical and radiochemical purity were determined using a Shimadzu LC2010 HPLC equipped with a Bioscan/Eckert and Ziegler radioactivity and ultraviolet detector (Column: Gemini NX, 5 μ, 250 × 4.6 mm, flow rate: 1 mL/min, mobile phase: 50% MeCN:H_2_O, 50 mM NH_4_HCO_3_, pH: 10, t_R_: 5.75 ± 0.1 min).

#### pH test

The pH of the formulated dose of [^11^C]COU was analyzed by placing a sample of the dose on a pH indicator strip. The indicator was then compared to the provided reference to determine the pH.

#### Filter integrity test

The sterile filter attached to the final dose vial was removed from the hot-cell. The filter and needle were then attached to a nitrogen supply with a pressure regulator. The needle was submerged in water and the nitrogen pressure was slowly increased. The pressure is increased until bubbles appear in the water (the bubble point). This pressure is then recorded.

#### Endotoxin test

Endotoxins were measured using a Charles River EndoSafe Portable Testing System. This is performed by adding a small amount of the formulated [^11^C]COU dose into the reservoir on the PTS cartridge to detect the amount of endotoxins in the sample.

#### Sterility test

Fluid thioglycolate media (FTM) and soybean casein digest agar media (SCDM) culture tubes were utilized for evaluation of the sterility of [^11^C]COU doses. The culture tubes were inoculated with dose samples then incubated at 20–25 °C (FTM) or 30–35 °C (SCDM) for 14 days alongside both positive and negative controls. The culture tubes were inspected visually at 3-, 8-, and 14-days post-inoculation. Sterility was confirmed based on the visual determination of turbidity in the samples based on both the positive and negative controls.

### Biodistribution and dosimetry

Male (n = 2) and female (n = 2) Sprague Dawley (SD) rats weighing 161–251 g were anesthetized using isoflurane and subsequently injected with 5.55–18.5 MBq of [^11^C]COU via femoral vein. Pressure was applied to the injection site to stop bleeding. The rats were allowed to recover from the anesthetic and resume normal activity. Each animal was sacrificed by decapitation at either 10, 20, 60, or 120 min post injection. Tissues and fluids were excised, homogenized, and counted for radioactivity in an auto-gamma counter to determine dosimetry and biodistribution. The results were corrected for radioactive decay and expressed in terms of percent-injected dose per gram of tissue (%ID/g) (see Supplementary Information and Supplementary Table 1 for more information).

The biodistribution data was used to estimate human internal radiation dosimetry, calculated from rat biodistribution using the MIRD formalism (see Supplementary Information and Table 2 for more information).

### Pharmacology and toxicology studies

Pharmacology and Toxicology studies were conducted at the Michigan State University In Vivo facility in Sprague Dawley rats. Rodents were housed individually and given free access to rodent chow and water. Animals were housed individually in plastic solid-bottom cages with aspen bedding throughout the duration of the study. In addition, animals had access to fresh water and were provided standard rodent chow in sufficient amounts to ensure ad libitum consumption. Sprague-Dawley rats were obtained from Charles River Laboratories and after approx. 1 week of acclimation, body weights were recorded [184–335 grams (males) or 200–249 grams (females)] at the time of dosing initiation. Light anesthesia was carried out using isoflurane, and then blood (~1 mL total) was collected into K_3_EDTA and serum tubes and was then processed for clinical chemistry and complete blood count (CBC) baseline measurements.

The animals were given either a single *i.v*. injection of 86 μg/kg of COU (100x the expected dose for PET imaging study) (18 male, 18 female) or 5% ethanol in saline vehicle (4 male, 4 female) and sacrificed either 2–4- or 15-days post-injection to look for acute or sub-acute effects, respectively. Throughout the study, food intake was periodically monitored. Additional blood samples were collected just prior to sacrifice for repeat CBC analysis. Following euthanasia, terminal body weights were obtained. Various tissues of interest (i.e. heart, kidneys, liver, and brain) were measured and fixed in 10% neutral-buffered formalin. Slides were prepared, stained with standard Hematoxylin and Eosin, and reviewed by a qualified veterinary pathologist.

## Conclusions

In this work, the radiosynthesis of [^11^C]COU has been validated, providing formulated product in acceptable yields, purities, molar activities, and sterility suitable for routine clinical use. Dosimetric studies have determined that, at a dose of 666 MBq, human subjects can receive two doses of [^11^C]COU without exceeding safe dose limits. From our pharmacology and toxicology studies we concluded that the maximum tolerated dose of COU was ≥86 μg/kg in Sprague Dawley rats. Human estimated maximum dose was set to ≥14 μg/kg based on the rodent studies, and the set dose for clinical trials is ≤0.14 μg/kg, 100-fold below the estimated maximum dose. From these preclinical studies, it can be concluded that [^11^C]COU is safe for translation into clinical studies. To this end, our lab has recently begun first-in-human studies using [^11^C]COU under the approval of the US Food and Drug Administration and the Michigan Medicine Institutional Review Board. These clinical studies are ongoing, and will be reported out in due course.

## Supplementary information

Supplementary Information

## Data Availability

Data is provided within the manuscript or supplementary information.

## Animal studies

Dosimetry studies were conducted under the approval of the University of Michigan (UM) Institutional Animal Care and Use Committee (IACUC), while Pharm-Tox studies were completed at the In Vivo Core under the approval of the Michigan State University (MSU) IACUC. Both UM and MSU are accredited by AAALAC International.

## Abbreviations

Bq: Becquerel
Kg: kilogram
IND: Investigational New Drug
FDA: Food and Drug Administration
IRB: Institutional Review Board
LOAEL: Least observable adverse effect level
NOAEL: no observable adverse effect level
GE: General Electric
CO_2_: carbon dioxide
MeOTf: methyl triflate
HPLC: high pressure liquid chromatography
mL: milliliter
Ci: curie
mmol: millimole
EU: endotoxin unit
MIRD: medical internal radiation dose
CBC: complete blood count
N/D: not detected
N/A: not applicable
