# Supplementary material for: IND-enabling preclinical studies of [11C]COU, a trapped metabolite PET radiotracer for monoamine oxidase-B
Source: Med Chem Res. 2025 Nov 5;34(12):2537–46. doi: 10.1007/s00044-025-03496-0 (PMC12622565; doi:10.1007/s00044-025-03496-0)
Supplement: Supplementary file 1 — Supplementary Information [file 44_2025_3496_MOESM1_ESM.pdf]

IND Enabling Preclinical Studies of [^{11}C]COU, a Trapped Metabolite Radiotracer for Monoamine Oxidase-B

Madison Frazier,[1,2] Tanpreet Kaur,[2] Jenelle Stauff,[2] Wade P. Winton,[2] Bradford D. Henderson,[2] Alexandra S. Dumond,[2] Xia Shao,[2] David M. Raffel,[2] Kirk A. Frey,[2] Michael R. Kilbourn,[2] Allen F. Brooks,[2] Peter J. H. Scott[1,2,3]*

[1] Department of Medicinal Chemistry, College of Pharmacy, University of Michigan, Ann Arbor, MI 48109.

[2] Department of Radiology, University of Michigan, Ann Arbor, MI 48109.

[3] Department of Pharmacology, University of Michigan, Ann Arbor, MI 48109.

[*] Correspondence: PJHS (email: pjhscott@umich.edu; ORCID: 0000-0002-6505-0450)

Table of Contents

1. Chemistry Validation
   1.1. General Considerations
   1.2. Large Scale Synthesis of COU precursor
   1.3. Large Scale Synthesis of COU standard
2. In Vivo Dosimetry
   2.1. Biodistribution
   2.2. Human Dosimetry Estimates
3. References
4. ^{1}H NMR, ^{13}C NMR, HRMS, and HPLC spectra
   4.1. 4-methyl-7-(pyridine-4-yloxy)-2H-chromen-2-one (**2**)
   4.2. 4-methyl-7-((1,2,3,6-tetrahydropyridin-4-yl)oxy)-2H-chromen-2-one (**4**)
   4.3. 4-methyl-7-((1-methyl-1,2,3,6-tetrahydropyridin-4-yl)oxy)-2H-chromen-2-one (**5**)
5. In Vivo Pharmacology and Toxicology Reports

1. Chemistry Validation

1.1. General Considerations

All the chemicals were purchased from commercially available suppliers and used without purification. Automated flash chromatography was performed with Biotage Isolera Prime system. High-performance liquid chromatography (HPLC) was performed using a Shimadzu LC-2010A HT system equipped with a Bioscan B-FC-1000 radiation detector. ^{1}H and ^{13}C NMR spectra: Varian 400 apparatus (400 MHz for 1H NMR and 101 MHz for ^{13}C NMR), in DMSO-d6 or CDCl3 unless otherwise indicated, δ in ppm rel. to tetramethylsilane (δ = 0), J in Hz. Mass spectra were measured on an Agilent Q-TOF HPLC-MS or VG (Micromass) 70-250-S Magnetic sector mass spectrometer employing the electrospray ionization (ESI) method.

1.2. Large Scale Synthesis of COU precursor

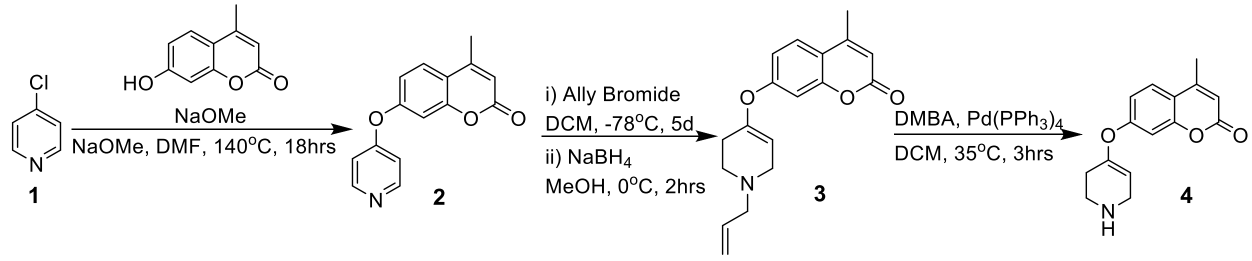

Supplemental Figure 1.

4-Methyl-7-(pyridin-4-yloxy)-2H-chromen-2-one (2):

To a round bottom flask, 4-methylumbelliferone (12 mmol, 1.2 eq) was dissolved in DMF (16 mL). This was then heated to 140°C for 15 minutes. 4-chloropyridine [1] (1.5 g, 1 eq) was added portion-wise to the reaction mixture. Reaction was allowed to stir at 140°C overnight. Next day, the reaction was quenched with sat. K_2CO_3 and subsequently extracted with ethyl acetate and water. Organic fraction was dried over Na_2SO_4. Product was purified by silica gel flash column chromatography (hexanes: ethyl acetate gradient).

Product collected in 12% yield.

^{1}H NMR (400 MHz; $CDCl_3$)/δ (ppm): 8.55 (2H, dd, J = 4.7, 1.6 Hz), 7.65 (1H, d, J = 8.5 Hz), 7.08-7.01 (2H, m), 6.94 (2H, dd, J = 4.7, 1.6 Hz), 6.27 (1H, s), 2.45 (3H, s)

^{13}C NMR (101 MHz; $CDCl_3$)/δ (ppm): 163.98, 160.51, 160.07, 157.57, 154.78, 152.04, 126.21, 116.82, 116.22, 113.94, 113.85, 109.76, 108.16, 24.43, 18.76

HRMS: calculated for [M]+1 (M = $C_{15}H_{11}NO_3$), 254.071, found 254.0828.

7-((1-Allyl-1,2,3,6-tetrahydropyridin-4-yl)oxy)-4-methyl-2H-chromen-2-one (3):
Prepared as previously described. Data agreed with that reported by Brooks, et al., 2020.[1]

4-Methyl-7-((1,2,3,6-tetrahydropyridin-4-yl)-oxy)-2H-chromen-2-one (COU) (4):
Prepared as previously described. Data agreed with that reported by Brooks, et al., 2020.[1]

^{1}H NMR (400 MHz; $CDCl_3$)/δ (ppm): 7.60 (1H, d, J = 8.7 Hz), 6.94 (1H, dd, J = 8.7, 2.4 Hz), 6.84(1H, d, J= 2.4, 6.09 (1H, s), 5.28 (1H, s), 3.49 (2H, s), 5.24(1H, s), 3.39 (2H, d, J = 3.1 Hz), 3.05 (2H, t, J = 5.9 Hz), 2.35 (3H, s)

^{13}C NMR (101 MHz; CD_3OD)/δ (ppm): 161.43, 159.41, 154.60, 153.75, 150.34, 126.20, 114.96, 114.56, 111.85, 108.55, 104.80, 42.48, 42.07, 25.93, 17.38

HRMS: calculated for [M]+1 (M = $C_{15}H_{15}NO_3$), 258.1023, found 258.1125.

HPLC Column: Gemini NX 5µ C18 110 A 250 x 4.6 mm

HPLC Buffer: 50 mM NH_4HCO_3 50% MeCN pH 10

Flow rate: 1.5 mL/min

1.3. Large Scale Synthesis of COU standard

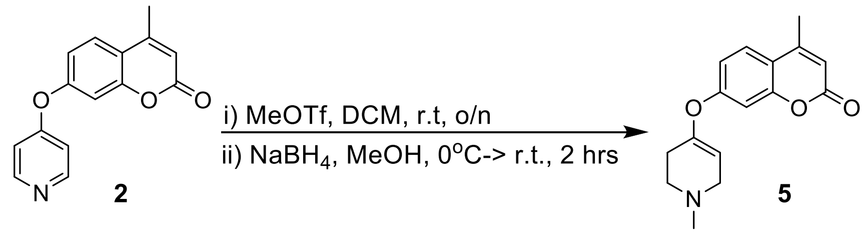

Supplemental Figure 2.

4-Methyl-7-((1-methyl-1,2,3,6-tetrahydropyridin-4-yl)oxy)-2H-chromen-2-one (5)

4-Methyl-7-(pyridin-4-yloxy)-2H-chromen-2-one (**2**) (1.0 g, 3.95 mmol) was dissolved in DCM (20 mL). Methyltriflate (1.36 mL, 11.85 mmol) was added to the reaction dropwise and stirred overnight. Next day, DCM was evaporated. Residue was dissolved in MeOH (20 mL) and cooled at 0 °C. Sodium borohydride (597.3 mg, 15.8 mmol) was added and stirred for 2 hrs. Next day, the reaction was quenched with sat. K_2CO_3 and subsequently extracted with ethyl acetate and water. Organic fraction was dried over Na_2SO_4. Product was purified by silica gel flash column chromatography (DCM: Methanol gradient).

Product collected in 35.3% yield (377.6 mg).

^{1}H NMR (400 MHz; $CDCl_3$)/δ (ppm): 7.54 (1H, d, J = 8.6 Hz), 7.04-6.96 (2H, m), 6.18 (1H, d, J = 1.3 Hz), 5.15-5.09 (1H, m), 3.30-3.22 (2H, m), 2.92 (2H, t, J = 5.9), 2.58 (3H, s), 2.49 (2H, s), 2.40 (3H, s)

^{13}C NMR (101 MHz; $CDCl_3$)/δ (ppm): 160.92, 158.73, 154.83, 152.32, 150.59, 125.91, 115.61, 115.33, 113.18, 106.53, 104.91, 52.50, 51.74, 44.68, 26.41, 18.78

HRMS: calculated for [M]+1 (M = $C_{16}H_{17}NO_3$), 272.1179, found 272.1281.

HPLC Column: Gemini NX 5µ C18 110 A 250 x 4.6 mm

HPLC Buffer: 50 mM NH_4HCO_3 50% MeCN pH 10

Flow rate: 1.5 mL/min

Manufacturing Process for [^{11}C[COU for Injection (API + Excipients) PET Drug Product

Unless otherwise stated, reagents and solvents were commercially available and used without further purification: Ethanol (200 proof, USP) was purchased from Decon Laboratories, Inc. Sodium chloride 0.9%, USP was sourced from Hospira. Other synthesis components were obtained as follows: Sterile vials were obtained from Hollister-Stier;C18 Sep-Pak's were purchased from Waters. Prior to use C18 Sep-Pak's were flushed with ethanol (10 mL) and sterile water (10 mL). Acetonitrile was purchased from Alfa Aesar.

Supplemental Figure 3: COU Manufacturing Flow Diagram

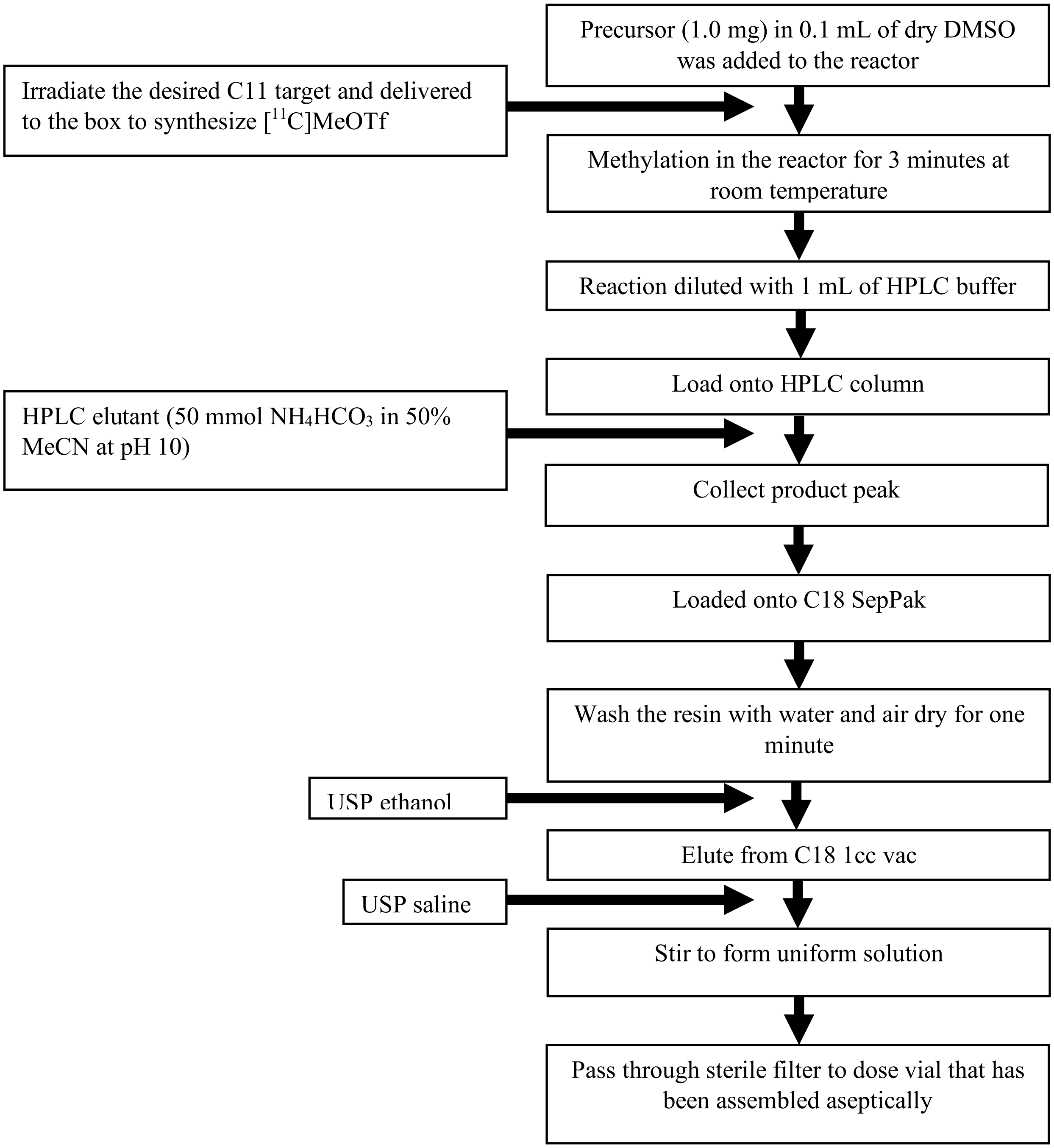

2. In Vivo Dosimetry

2.1. Biodistribution

For each timepoint, male (n=2) and female (n=2) Sprague Dawley (SD) rats weighing 161–251 g were anesthetized using isoflurane and subsequently injected with 5.55-18.5 MBq of [^{11}C]COU via femoral vein.[2] Pressure was applied to the injection site to stop bleeding. The rats were allowed to recover from the anesthetic and resume normal activity. Each animal was

sacrificed by decapitation at either 10, 20, 60, or 120 min post injection. Tissues and fluids were excised, homogenized, and counted for radioactivity in an auto-gamma counter to determine dosimetry and biodistribution. Brain samples included striatum, whole cerebral cortex, hippocampus, hypothalamus, and cerebellum. The results were corrected for radioactive decay and expressed in terms of percent-injected dose per gram of tissue (%ID/g) (Supplemental Table 1). Each data point represents the mean values from the four animals used for the experiment.

Supplemental Table 1. [^{11}C]COU biodistribution in rodents shown as %ID/g of tissue (average of n=4 per timepoint).

ORGANS	10 min	20 min	60 min	120 min
BRAIN	0.155	0.101	0.068	0.079
EYEBALLS	0.109	0.056	0.036	0.029
HEART	0.102	0.081	0.057	0.070
LUNG	0.195	0.107	0.049	0.035
LIVER	0.626	0.603	0.085	0.039
PANCREAS	0.212	0.172	0.072	0.067
SPLEEN	0.211	0.185	0.098	0.088
ADRENAL	0.252	0.354	0.211	0.218
KIDNEY	1.546	0.475	0.124	0.096
ADIPOSE	0.022	0.026	0.017	0.008
STOMACH	0.368	0.217	0.075	0.024
CONTENTS OF STOMACH	0.284	0.211	0.568	0.013
SMALL INTESTINE	0.819	0.777	0.939	0.219
CONTENTS OF SMALL INTESTINE	0.130	0.167	0.933	0.821
CAECUM	0.095	0.068	0.062	0.151
CONTENTS OF CAECUM	0.012	0.004	0.014	0.576
LARGE INTESTINE	0.126	0.114	0.069	0.052
CONTENTS OF LARGE INTESTINE	0.015	0.015	0.013	0.013
OVARY	0.168	0.229	0.084	0.059
UTERUS	0.129	0.084	0.060	0.058
MUSCLE	0.021	0.020	0.017	0.012

BONE	0.051	0.051	0.028	0.022
BLOOD	0.033	0.030	0.015	0.009
TESTES	0.101	0.052	0.037	0.025

2.2. Human Dosimetry Estimates

The biodistribution data was used to estimate human internal radiation dosimetry, calculated from rat biodistribution using the MIRD formalism Table 2.[3] Consistent with the short half-life of carbon-11 (20 min), dosimetry burden is estimated to be low. The bladder wall was estimated to receive the largest dose, 0.0452 mSv/MBq. Applying the guidelines of RDRCs operating in the United States, the dose limit for adults is defined as follows: 30 mSv per single injection for the whole body, lens of the eyes, red marrow, and gonads; 50 mSv per year for the whole body, lens of the eyes, red marrow, and gonads; 50 mSv per single injection for all other organs; and 150 mSv per year for all other organs (FDA 21 CFR 361.1). The estimated dosimetry for [^{11}C]COU allows administration of up to 2 x 666 MBq doses of [^{11}C]COU with an effective dose of 3.06 mSv, this falls well within the acceptable limits established by the RDRC.

3. References

(1) Brooks, A. F.; Mufarreh, A. J.; Shao, X.; Kaur, T.; Stauff, J.; Arteaga, J.; Kilbourn, M. R.; Scott, P. J. H. Improved Synthesis of [^{11}C]COU and [^{11}C]PHXY, Evaluation of Neurotoxicity, and Imaging of MAOs in Rodent Heart. *ACS Med. Chem. Lett.* **2020**, 11 (11), 2300–2304.

(2) Stabin, M. G.; Siegel, J. A. Physical Models and Dose Factors for Use in Internal Dose Assessment. *Health Phys.* **2003**, *85* (3), 294–310.

(3) Stabin, M. G. OLINDA/EXM 2—The Next-Generation Personal Computer Software for Internal Dose Assessment in Nuclear Medicine. *Health Phys.* **2023**, *124* (5), 397. https://doi.org/10.1097/HP.0000000000001682.

4. ^{1}H NMR, ^{13}C NMR, HRMS, and HPLC spectra

4.1. 4-Methyl-7-(pyridin-4-yloxy)-2H-chromen-2-one (2)

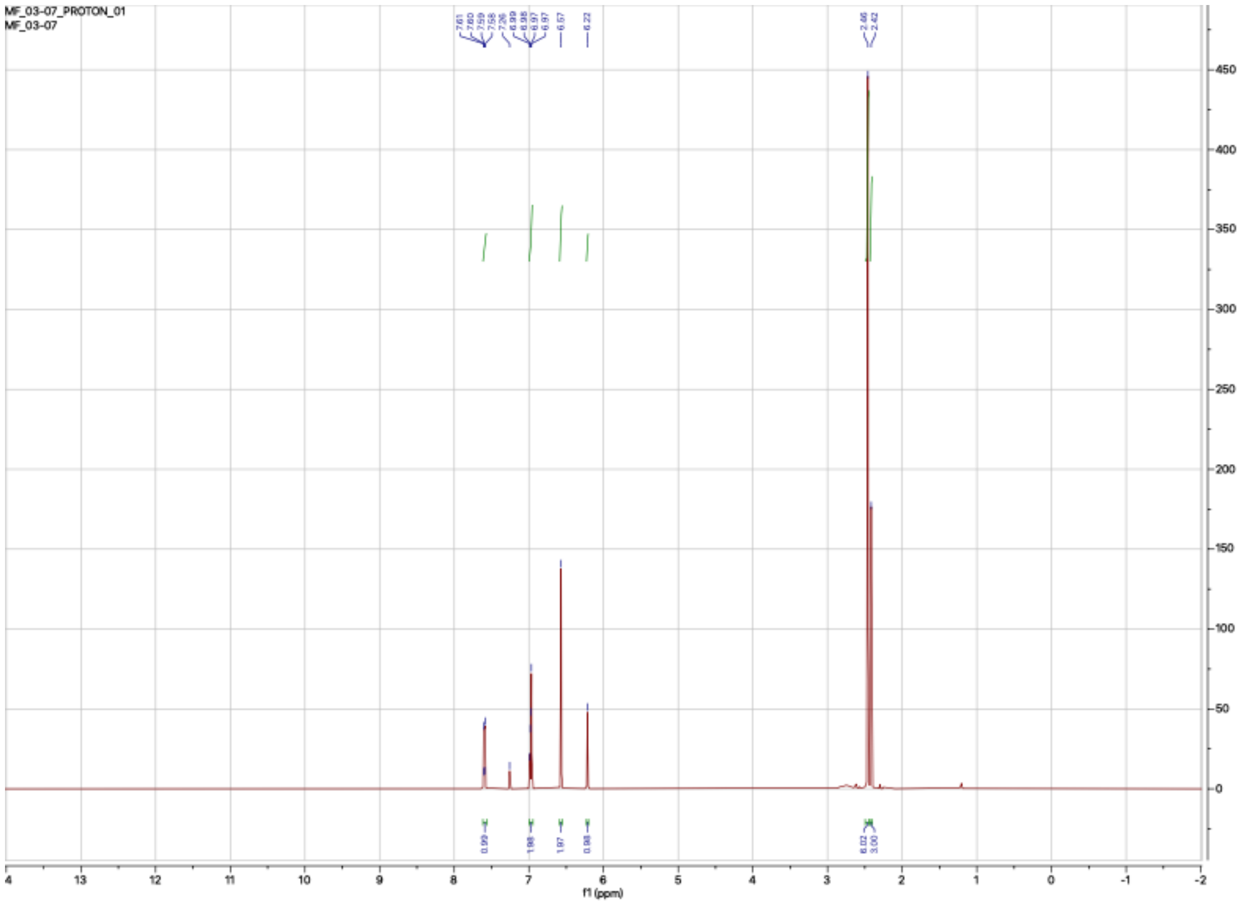

Supplemental Figure 4. ^{1}H NMR of 4-methyl-7-(pyridin-4-yloxy)-2H-chromen-2-one (**2**).

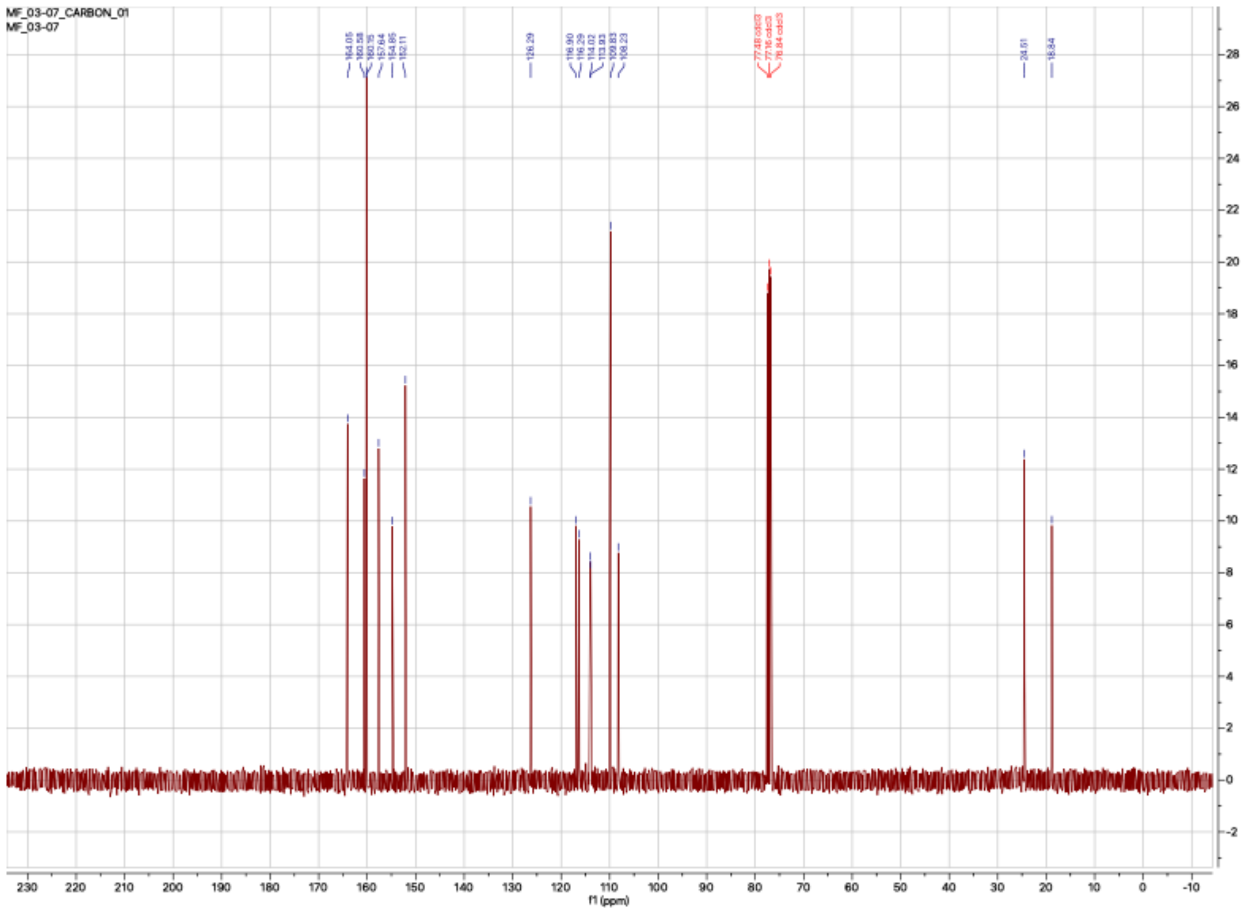

Supplemental Figure 5. ^{13}C NMR of 4-methyl-7-(pyridin-4-yloxy)-2H-chromen-2-one (**2**).

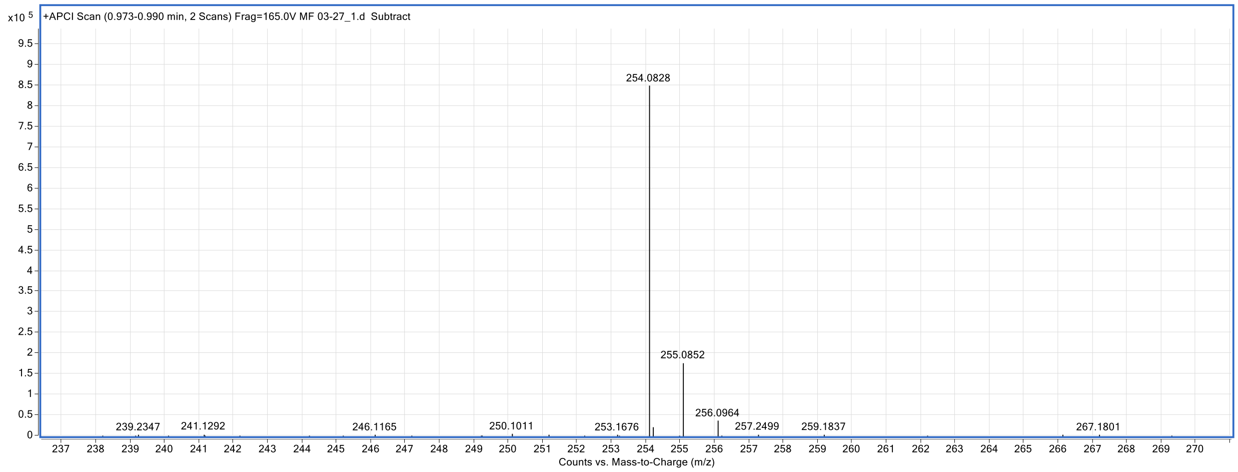

Supplemental Figure 6. HRMS of 4-methyl-7-(pyridin-4-yloxy)-2H-chromen-2-one (**2**).

4.2. 4-methyl-7-((1,2,3,6-tetrahydropyridin-4-yl)oxy)-2H-chromen-2-one (4)

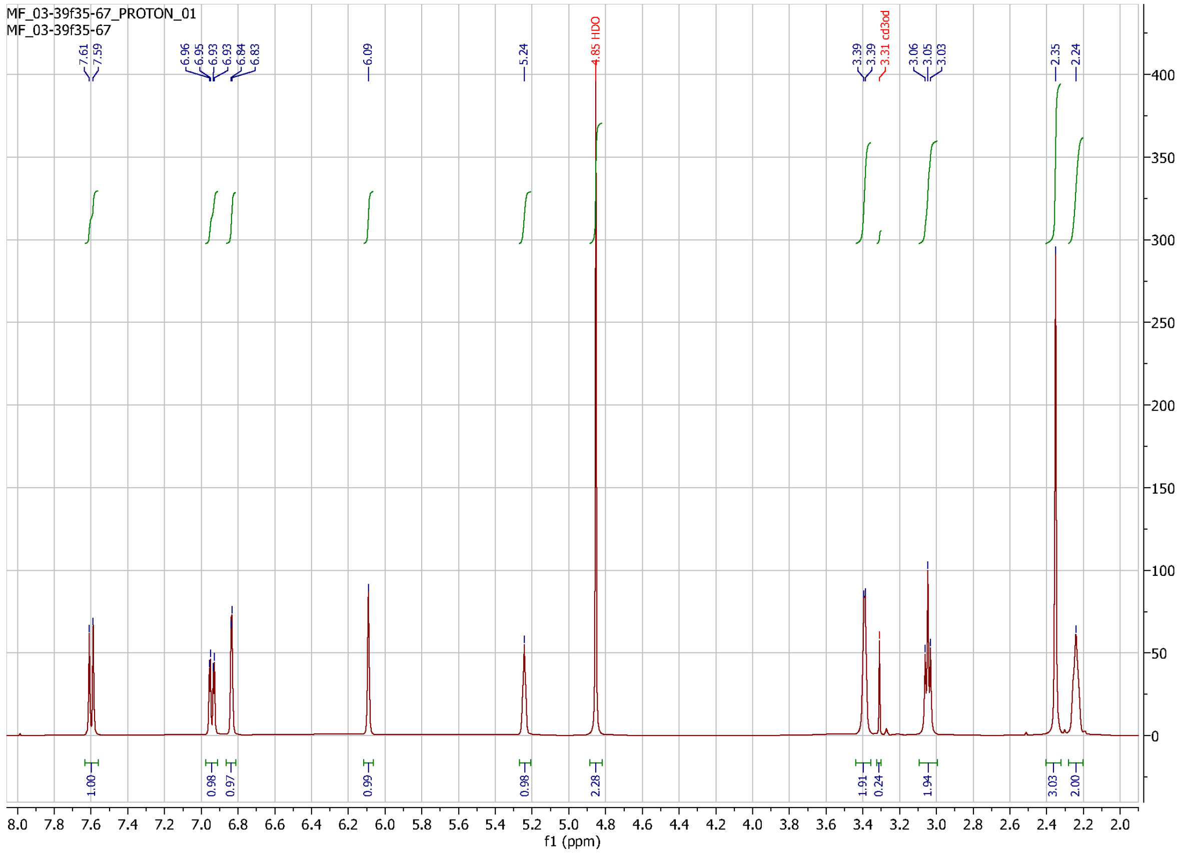

Supplemental Figure 7. ^{1}H NMR of 4-methyl-7-((1,2,3,6-tetrahydropyridin-4-yl)oxy)-2H-chromen-2-one (**4**).

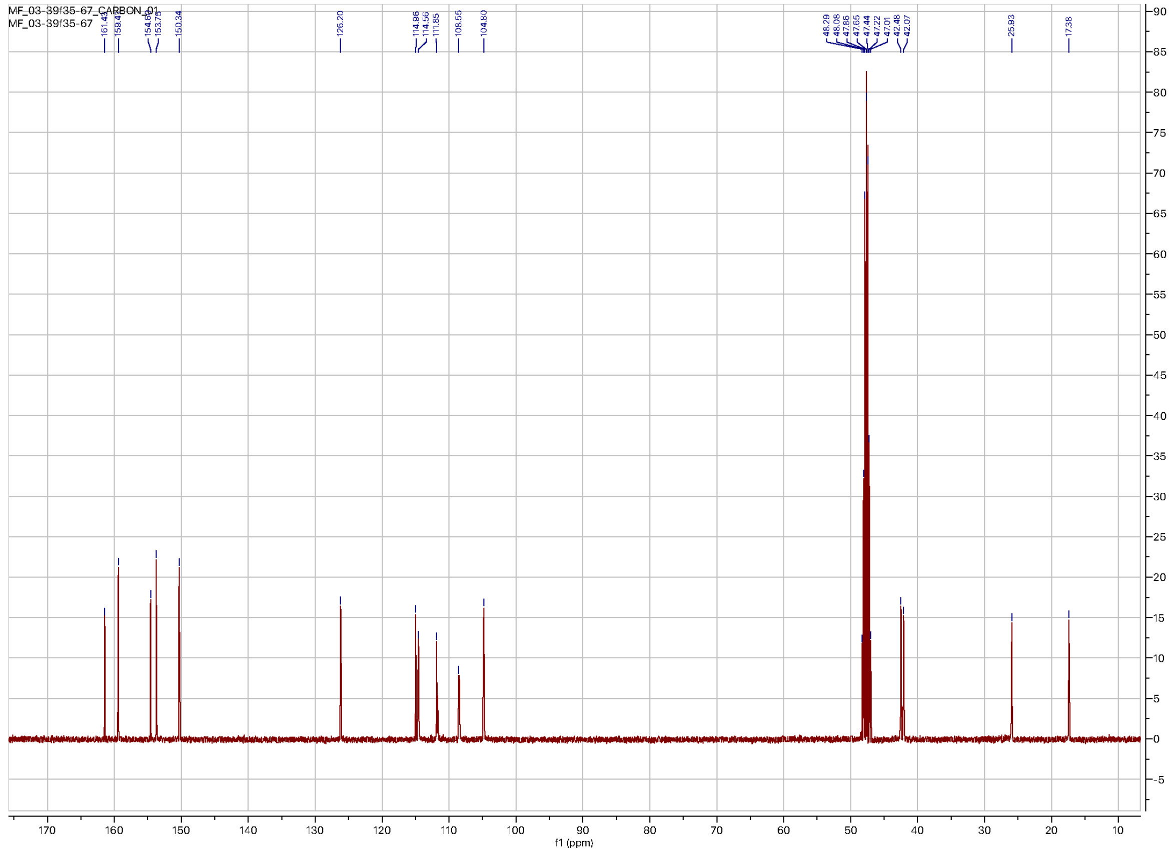

Supplemental Figure 8. ^{13}C NMR of 4-methyl-7-((1,2,3,6-tetrahydropyridin-4-yl)oxy)-2H-chromen-2-one (**4**).

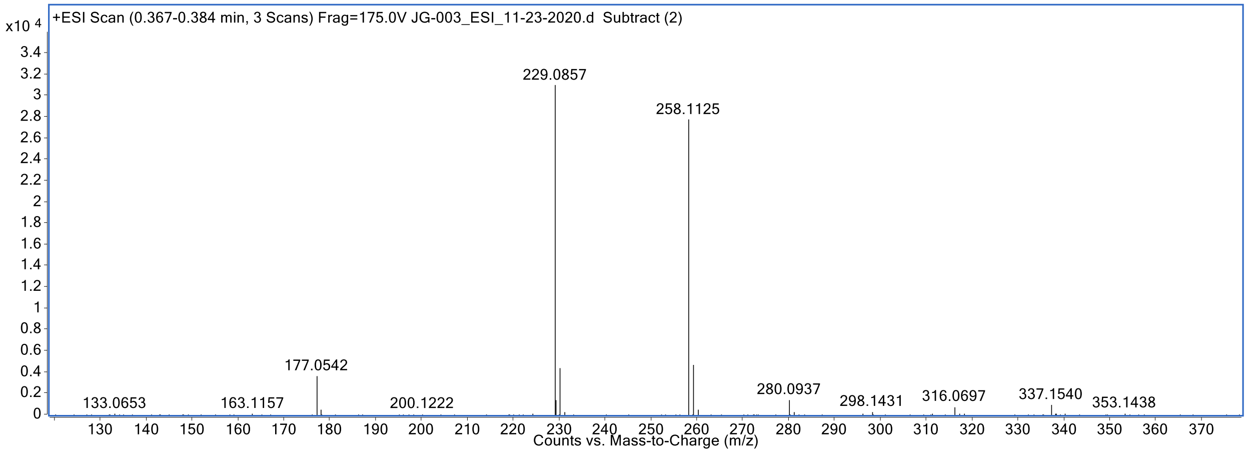

Supplemental Figure 9. HRMS of 4-methyl-7-((1,2,3,6-tetrahydropyridin-4-yl)oxy)-2H-chromen-2-one (**4**).

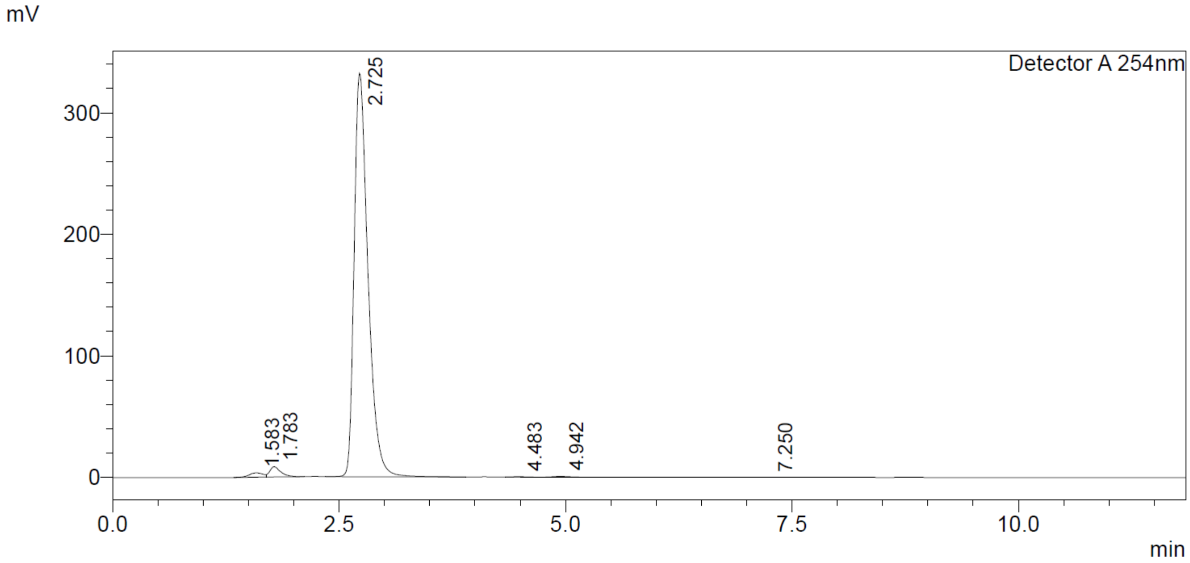

Peak#	Ret. Time	Area	Height	Area%
1	1.583	37100	3567	1.016
2	1.783	72642	8520	1.990
3	2.725	3524666	332042	96.567
4	4.483	3054	319	0.084
5	4.942	8452	644	0.232
6	7.250	4054	127	0.111
Total		3649969	345219	100.000

Supplemental Figure 10. Analytical HPLC of 4-methyl-7-((1,2,3,6-tetrahydropyridin-4-yl)oxy)-2H-chromen-2-one (**4**).

4.3 4-methyl-7-((1-methyl-1,2,3,6-tetrahydropyridin-4-yl)oxy)-2H-chromen-2-one (5)

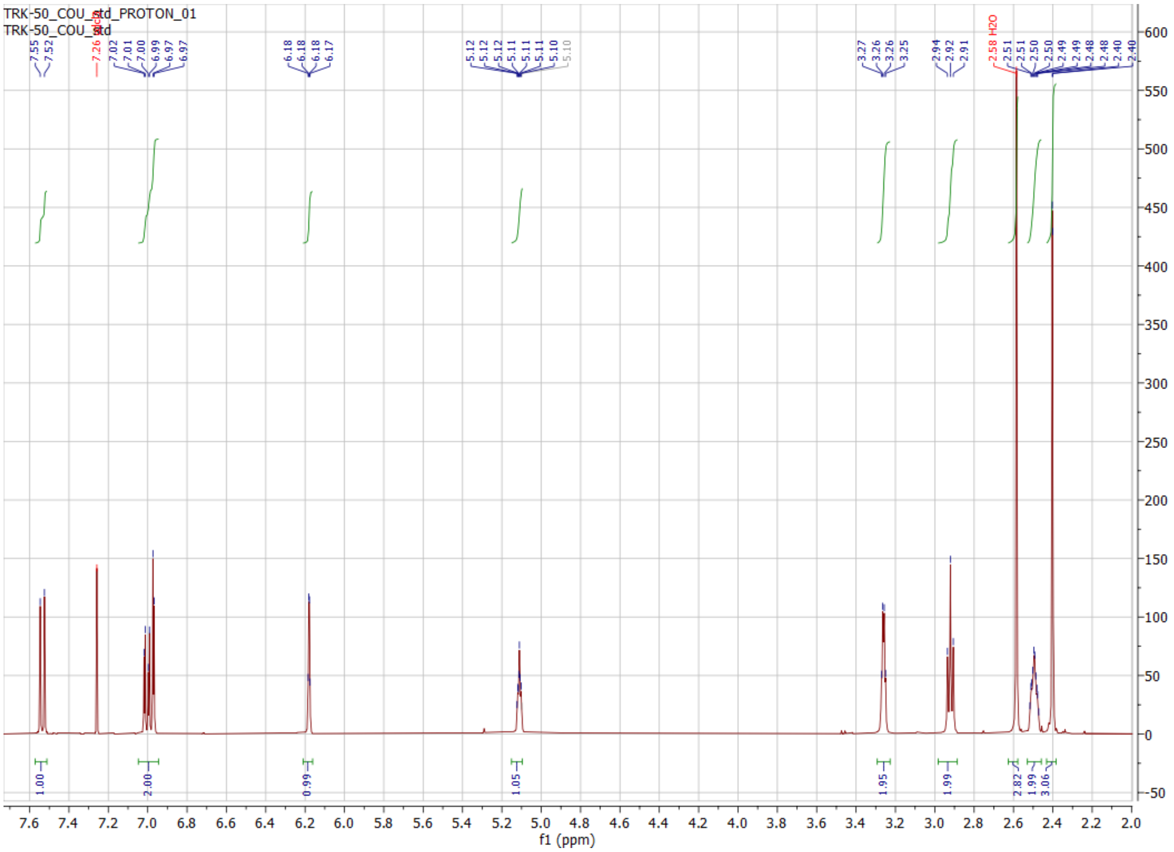

Supplemental Figure 11. ^{1}H NMR of 4-methyl-7-((1-methyl-1,2,3,6-tetrahydropyridin-4-yl)oxy)-2H-chromen-2-one (**5**).

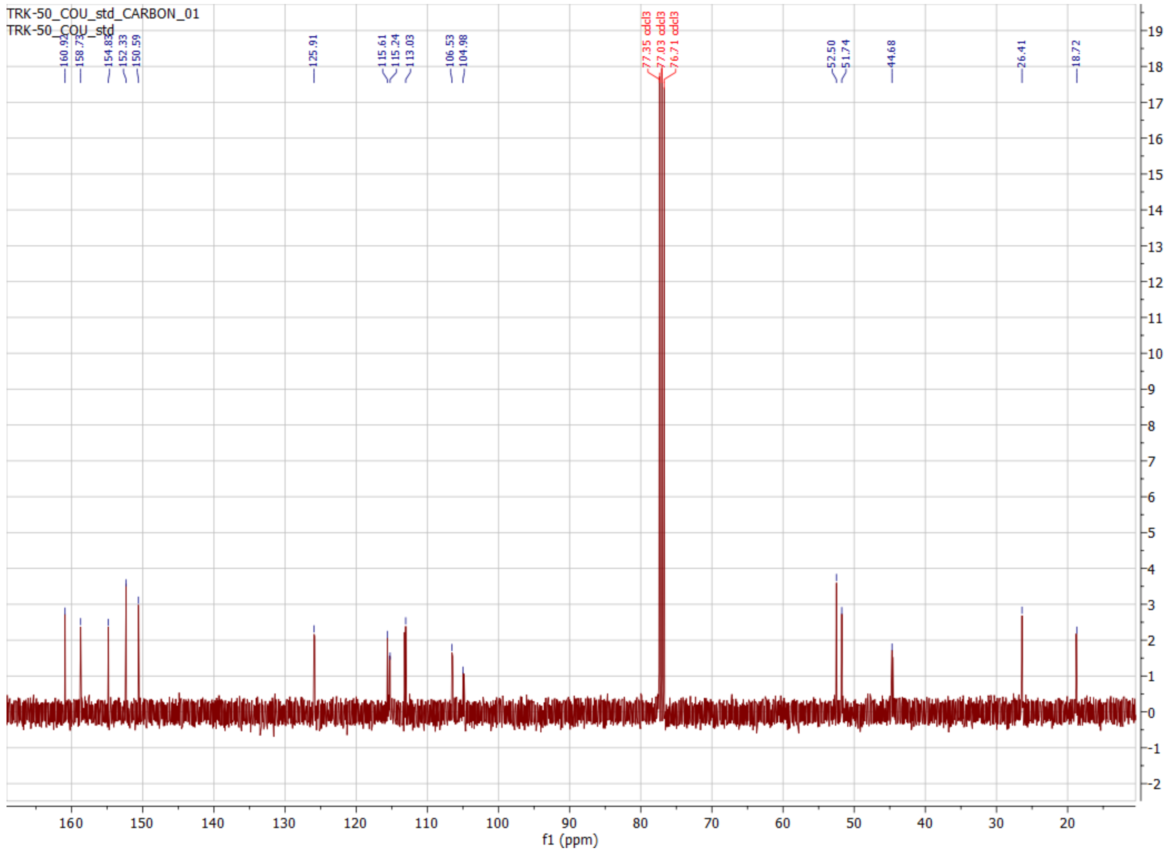

Supplemental Figure 12. ^{13}C NMR of 4-methyl-7-((1-methyl-1,2,3,6-tetrahydropyridin-4-yl)oxy)-2H-chromen-2-one (**5**).

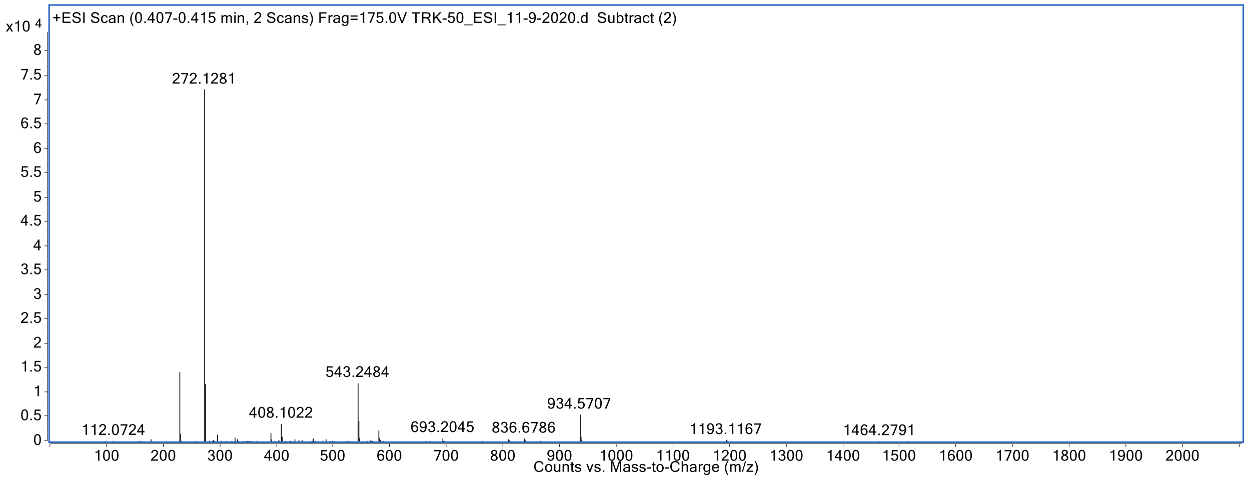

Supplemental Figure 13. HRMS of 4-methyl-7-((1-methyl-1,2,3,6-tetrahydropyridin-4-yl)oxy)-2H-chromen-2-one (**5**).

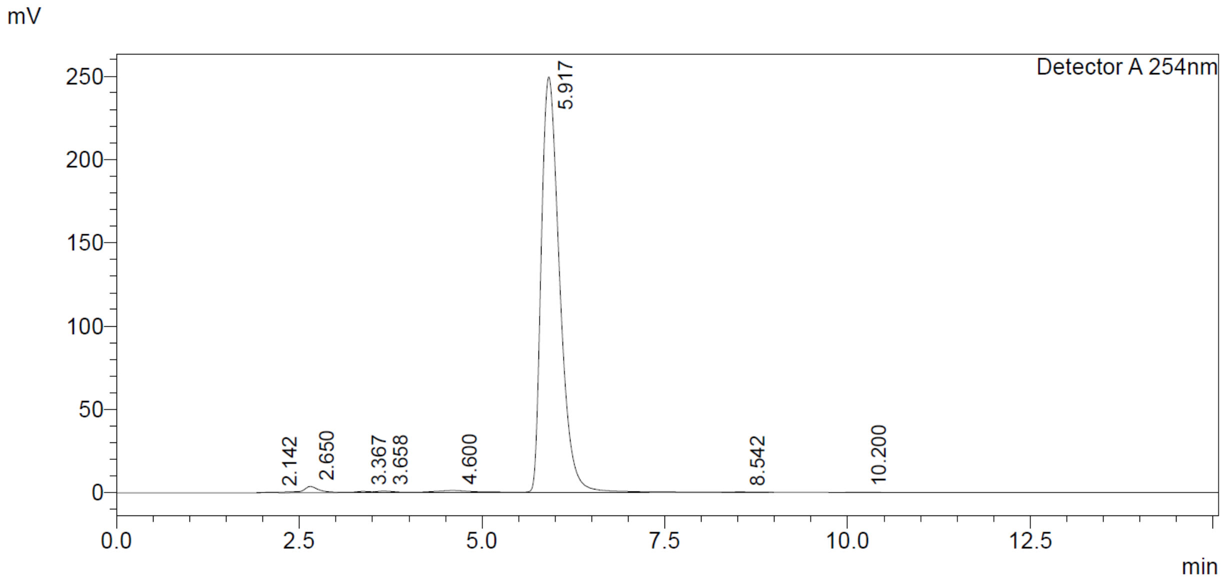

Peak#	Ret. Time	Area	Height	Area%
1	2.142	3933	359	0.087
2	2.650	61184	3768	1.353
3	3.367	10725	751	0.237
4	3.658	16730	956	0.370
5	4.600	50855	1264	1.125
6	5.917	4370750	249435	96.687
7	8.542	2947	161	0.065
8	10.200	3388	165	0.075
Total		4520511	256858	100.000

Supplemental Figure 14. Analytical HPLC of 4-methyl-7-((1-methyl-1,2,3,6-tetrahydropyridin-4-yl)oxy)-2H-chromen-2-one (**5**).

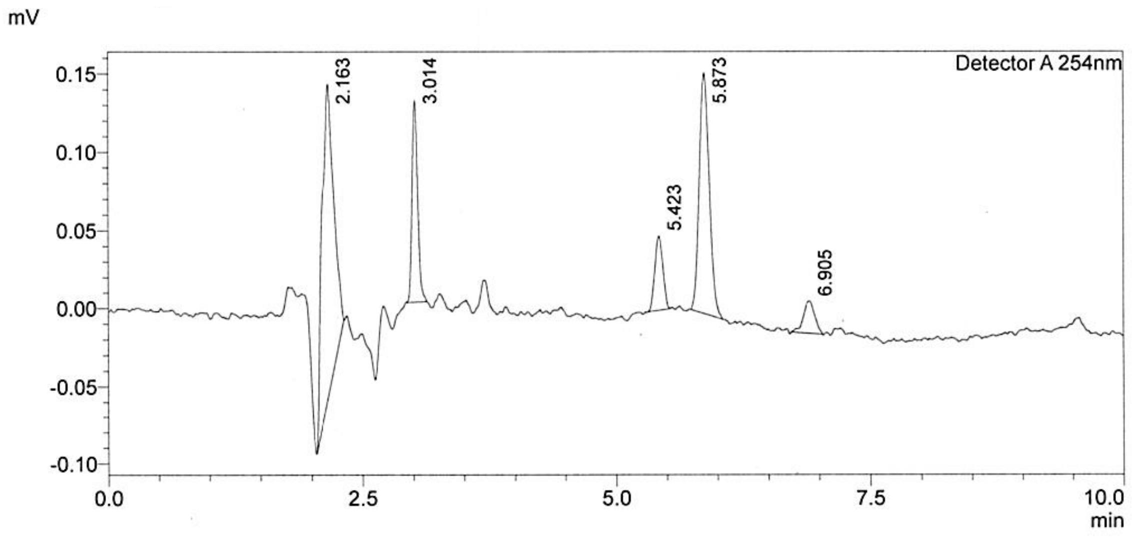

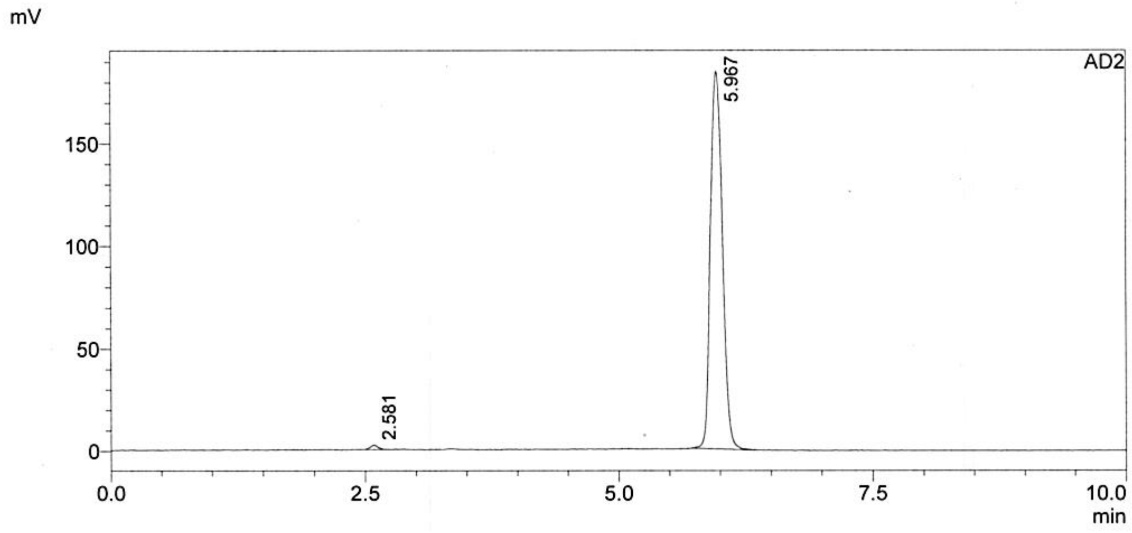

Peak#	Ret. Time	Area	Height	Conc.	Area%
1	2.163	1664	200	45.922	45.922
2	3.014	495	129	13.668	13.668
3	5.423	259	48	7.159	7.159
4	5.873	1044	154	28.806	28.806
5	6.905	161	21	4.445	4.445
Total		3624	551		100.000

AD2

Peak#	Ret. Time	Area	Height	Conc.	Area%
1	2.581	11323	2140	0.000	0.747
2	5.967	1503911	184207	0.000	99.253
Total		1515234	186347		100.000

Supplemental Figure 15. Analytical HPLC of [^{11}C]COU.

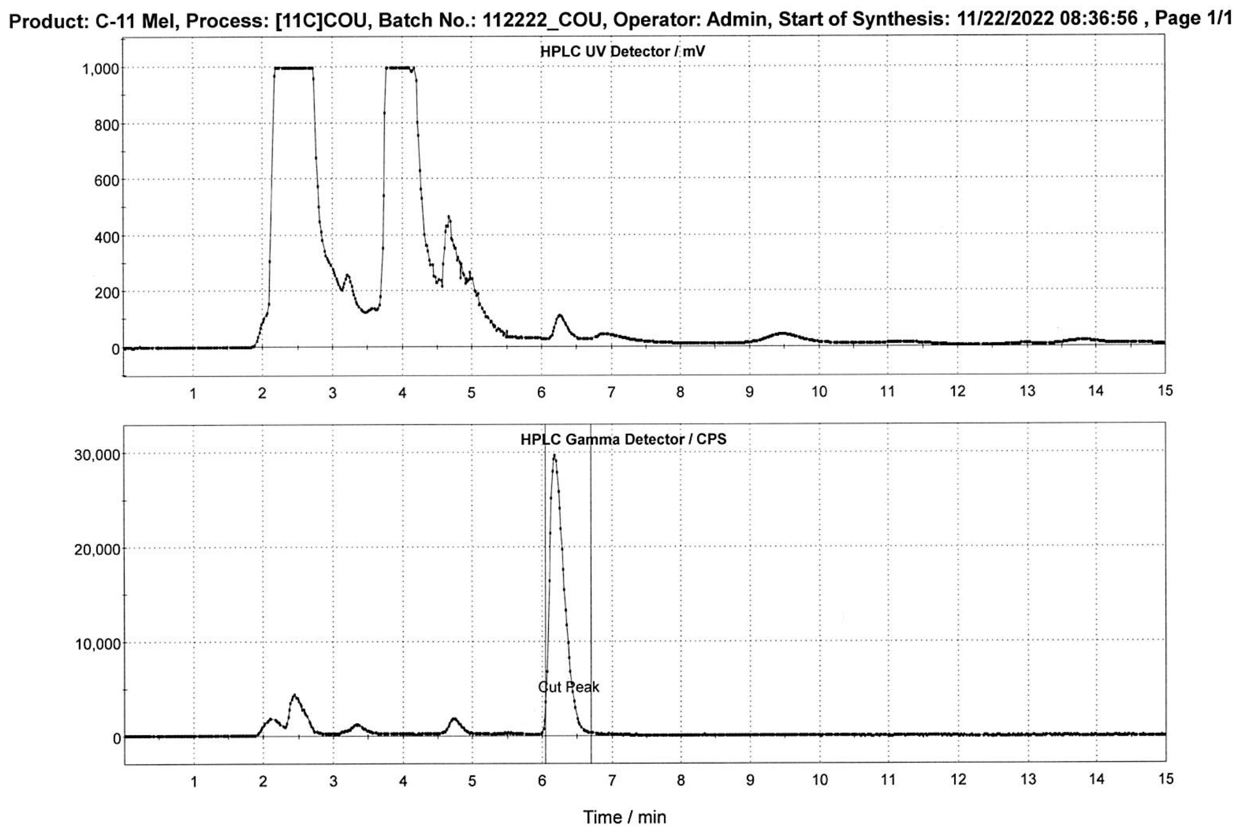

Supplemental Figure 16. Semi-preparative HPLC of [^{11}C]COU.

Single Dose IV UM_COU Rat Study
Study Number: 2021101

Michigan State University In Vivo Facility
East Lansing, Michigan 48824

Sponsor: **Peter J. H. Scott, PhD**
Division of Nuclear Medicine / Department of Radiology
University of Michigan Medical School

Sponsor Contact: **Peter J.H. Scott, PhD**

APPROVAL SIGNATURES

The following individuals confirm that this report accurately represents their interpretation of the data:

Date: 2022.03.15
18:48:07 -04'00'

3/15/2022

Teresa Krieger-Burke, DVM, PhD | Date

TABLE OF CONTENTS

APPROVAL SIGNATURES 2
TABLE OF CONTENTS 3
1. OBJECTIVE 4
2. MATERIALS AND METHODS 4
  2.1. Test Article 4
  2.2. Dosing Formulation Preparation 4
  2.3. Animal Information 4
  2.4. Experimental Schedule 5
  2.5. Description of Experimental Procedure 5
  2.6. Statistical Analyses 7
3. RESULTS 7
  3.1. Clinical Observations 7
  3.2. Body Weight 7
  3.3. Food Consumption 8
  3.4. Organ Weights and Necropsy Gross Findings 8
  3.5. Hematology 10
  3.6. Clinical Biochemistry 12
  3.7. Histopathology 18
4. CONCLUSION 18
Appendix I. Individual Body Weight over Time 19
Appendix II. Individual Organ Weights 20
Appendix III. Individual Hematology Values 22
Appendix IV. Individual Biochemistry Values 26
Appendix V. Individual Microscopic Findings 34

Single Dose IV UM_COU Rat Study

1. OBJECTIVE

The objective of this study was to investigate the toleration of a single intravenous dose of UM_COU in male and female rats.

2. MATERIALS AND METHODS

2.1. Test Article

Compound:	UM_COU
Lot Number:	not applicable
Active Moiety (used for dose calculations):	100% assumed
Vehicle:	not applicable
Dose:	86 µg/kg
Dose Volume:	0.86 mL/kg
Dose Route of Administration:	intravenous
Storage Conditions:	refrigerated, protected from light

2.2. Dosing Formulation Preparation

Dosing formulation was provided by the Sponsor ready-to-dose. The formulation was stored under refrigerated conditions, protected from light, until the time of use. Formulations were used within 10 days of delivery.

2.3. Animal Information

Ten (10) male and 10 female Sprague-Dawley rats were obtained from Charles River Laboratories and weighed 184-241 grams (males) or 200-229 grams (females) at the time of dosing initiation. Animals were individually housed in solid bottom cages with aspen bedding for the duration of the study. All animals had free access to standard rodent chow and fresh water throughout the study. Animals were maintained on an automated 12/12 hour light/dark cycle with 7:00am as the start of the light phase.

This study was conducted in accordance with the current guidelines for animal welfare (Guide for the Care and Use of Laboratory Animals, 8th Ed., 2011). The procedures used in this study have been reviewed and approved by the Institutional Animal Care and Use Committee.

2.4. Experimental Schedule

Animals were assigned to an experimental schedule according to Table 1 below.

Table 1. Treatment Schedule

Group	No. of Animals	Animal Numbers	Sex	Treatment	Scheduled Necropsy Day
1	5	101-105	M	UM_COU 86 µg/kg	Day 15
2	5	201-205	M	UM_COU 86 µg/kg	Day 4
3	5	301-305	F	UM_COU 86 µg/kg	Day 15
4	5	401-405	F	UM_COU 86 µg/kg	Day 4

Test Subject Arrival Date: June 29, 2021

In-Life Start Date (Study Day 1): July 6, 2021

Monitor End Date (Completion of In-Life Phase): July 20, 2021

Data Collection Rooms: B-23 and B-346, Life Sciences building, MSU

Clinical Observations: pre-dose and daily thereafter

Body Weights: prior to dosing and at time of necropsy

Food Consumption: Study Day -1 (baseline), and Days 3, 7, and 15

Serum Biochemistry and Complete Blood Counts (CBC): prior to dosing (baseline) and at scheduled necropsy (Day 4: Groups 2 & 4; Day 15: Groups 1 & 3)

Organs Collected and Weighed: Heart, liver, kidney, spleen, and brain

Disposition of Animals at Study Completion: Euthanized at the time of the final blood collection and carcasses incinerated

2.5. Description of Experimental Procedure

Ten (10) male and 10 female Sprague-Dawley rats were used on study. Animals were received from the supplier and housed in room B-23 in the vivarium of the Life Sciences building at Michigan State University. Animals were individually housed in plastic solid bottom cages with aspen bedding throughout the duration of the study. Animals had free access to standard rodent chow and fresh water and were maintained on an automated 12/12 hour light/dark cycle with 7:00am as the start of the light phase.

Animals were acclimated at least 1 week. The study was conducted in two cohorts to facilitate timing of blood sample delivery to the Veterinary Diagnostic Laboratory on Day 1 (dosing day). Groups 1 and 3 were dosed on July 6, 2021. Groups 2 and 4 were dosed on July 12, 2021. The day of dosing of each individual animal was considered Study Day 1 for that animal. Animals were lightly anesthetized using isoflurane in oxygen on Study Day 1. Blood (~1mL total) was collected into K_3EDTA and serum tubes, and processed for respective complete blood count (CBC) and clinical chemistry baseline measurements. The test article formulation was then delivered as a single intravenous bolus and animals were recovered from anesthesia.

Clinical observations were recorded daily. Food consumption was measured on Day -1 (baseline) and on Days 3, 7, and 15.

Groups 2 and 4 were euthanized on Day 4 and Groups 1 and 3 on Day 15. Terminal body weights were recorded. Terminal blood samples were collected for hematology and biochemistry analyses. Animals were euthanized by thoracotomy immediately following the final blood collection. A limited gross necropsy was performed, and heart, liver, kidneys, spleen, and brain removed, weighed, and fixed in 10% neutral-buffered formalin (see Gross Pathology & Histopathology section below). Slides were prepared, stained with standard Hematoxylin & Eosin, and reviewed by a veterinary pathologist.

2.5.1. Hematology

Blood samples containing K_3EDTA as an anticoagulant were analyzed by the MSU Veterinary Diagnostic Laboratory (VDL) for hematology parameters including:

red cell count	red cell distribution width
hemoglobin	platelet count
hematocrit	mean platelet volume
mean corpuscular volume	white cell count
mean corpuscular hemoglobin	white cell differential
mean corpuscular hemoglobin concentration	

2.5.2. Biochemistry

Blood samples were collected into clot tubes. Serum was analyzed by the MSU VDL for biochemistry parameters including:

alanine aminotransferase	chloride	phosphorus
albumin	cholesterol	potassium
alkaline phosphatase	creatinine	protein (total)
aspartate aminotransferase	globulin (calculated)	sodium
bilirubin (total)	glucose	urea nitrogen (BUN)
calcium	magnesium	

2.5.3. Gross Pathology and Histopathology

The following tissues were evaluated grossly in all animals:

adrenal	large & small intestine	spleen
brain	liver	stomach
heart	lung	thymus
kidney	pancreas	urinary bladder

Representative samples of the following tissues were collected from all rats, weighed, fixed in 10% buffered formalin, embedded in paraffin, sectioned, and stained with hematoxylin and eosin:

Heart	Spleen	Liver	Kidney (L&R weighed together)	Brain

2.6. Statistical Analyses

Data were collected in Excel. Graphic interpretation of the data was performed using GraphPad Prism 9.2.0. Statistical comparisons of body weight, organ weights, hematology, and clinical chemistry parameters were made using Kruskal-Wallis one-way analysis of variance followed by post-hoc testing using Dunn's multiple comparisons test. In all cases, a $p<0.05$ was considered statistically significant. Numbers comparing groups on graphs indicate the corresponding p-values.

3. RESULTS

3.1. Clinical Observations

There were no drug-related clinical findings. All animals remained within normal limits throughout the study.

3.2. Body Weight

There were no adverse drug-related effects on body weight gain in males or females (Figure 1).

Figure 1. Mean Body Weight over Time

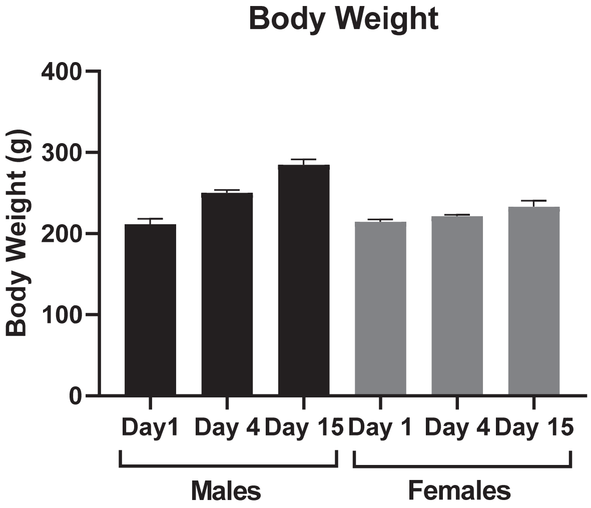

		Day 1	Day 4	Day 15
Males	Mean	211.7	250.3	284.9
	SEM	6.7	3.4	6.7
	n	10	5	5
Females	Mean	214.4	221.6	233.3
	SEM	3.3	1.7	7.3
	n	10	5	5

3.3. Food Consumption

Average daily food consumption of male animals remained similar over time as did average daily food consumption in females (Table 2).

Table 2. Average Daily Food Consumption (grams) over Time

	Animal No.	Baseline	Day 3	Day 7	Day 15
Males Day 4 cohort	201	19.9	21.6	NA	NA
	202	21.7	24.6	NA	NA
	203	20.7	23.1	NA	NA
	204	20.7	20.7	NA	NA
	205	23.5	24.8	NA	NA
	**Mean**	**21.3**	**23.0**		
	**SEM**	**0.6**	**0.8**		
Males Day 15 cohort	101	21.7	21.2	24.1	25.4
	102	19.3	20.6	23.1	22.9
	103	19.2	20.5	22.1	23.1
	104	19.8	19.9	23.6	23.2
	105	20.5	19.0	22.8	24.3
	**Mean**	**20.1**	**20.2**	**23.1**	**23.8**
	**SEM**	**0.5**	**0.4**	**0.3**	**0.5**
Females Day 4 cohort	401	16.9	16.6	NA	NA
	402	17.4	17.9	NA	NA
	403	16.4	16.6	NA	NA
	404	16.4	19.6	NA	NA
	405	18.2	15.9	NA	NA
	**Mean**	**17.1**	**17.3**		
	**SEM**	**0.3**	**0.6**		
Females Day 15 cohort	301	16.1	19.8	19.7	15.5
	302	17.2	12.9	18.4	18.6
	303	19.0	16.7	19.7	18.6
	304	14.7	15.6	16.8	16.3
	305	17.8	15.2	18.5	17.3
	**Mean**	**17.0**	**16.0**	**18.6**	**17.3**
	**SEM**	**0.7**	**1.1**	**0.5**	**0.6**

3.4. Organ Weights and Necropsy Gross Findings

There were no significant gross necropsy findings.

Organ weights were comparable within each sex between Day 4 and Day 15 (Tables 3 to 5).

Table 3. Terminal Organ Weights

Organ Weight (g)						
Group		Brain	Heart	Liver	Spleen	Kidneys (combined)
Males, Day 4	Mean	**1.733**	**0.983**	**9.570**	**0.681**	**1.752**
	SEM	**0.048**	**0.052**	**0.339**	**0.045**	**0.038**
Males, Day 15	Mean	**1.737**	**1.059**	**11.250**	**0.629**	**2.089**
	SEM	**0.019**	**0.018**	**0.320**	**0.016**	**0.072**
Females, Day 4	Mean	**1.849**	**0.926**	**7.802**	**0.604**	**1.471**
	SEM	**0.069**	**0.028**	**0.211**	**0.044**	**0.038**
Females, Day 15	Mean	**1.789**	**0.920**	**8.025**	**0.552**	**1.496**
	SEM	**0.028**	**0.087**	**0.293**	**0.036**	**0.060**

Table 4. Terminal Organ Weight to Body Weight Ratios

Organ Weight:Body Weight						
**Group**		**Brain:BW Ratio**	**Heart:BW Ratio**	**Kidneys:BW Ratio**	**Spleen:BW Ratio**	**Liver:BW Ratio**
Males, Day 4	Mean	**0.0069**	**0.0039**	**0.0070**	**0.0027**	**0.0382**
	SEM	**0.0002**	**0.0002**	**0.0001**	**0.0001**	**0.0009**
Males, Day 15	Mean	**0.0061**	**0.0037**	**0.0073**	**0.0022**	**0.0394**
	SEM	**0.0002**	**0.0001**	**0.0002**	**0.0001**	**0.0005**
Females, Day 4	Mean	**0.0083**	**0.0042**	**0.0066**	**0.0027**	**0.0352**
	SEM	**0.0003**	**0.0001**	**0.0002**	**0.0002**	**0.0008**
Females, Day 15	Mean	**0.0077**	**0.0039**	**0.0064**	**0.0024**	**0.0346**
	SEM	**0.0002**	**0.0002**	**0.0003**	**0.0001**	**0.0005**

Table 5. Terminal Organ Weight to Brain Weight Ratios

Organ Weight:Brain Weight					
**Group**		**Heart:Brain Wt Ratio**	**Kidneys:Brain Wt Ratio**	**Spleen:Brain Wt Ratio**	**Liver:Brain Wt Ratio**
Males, Day 4	Mean	**0.57**	**1.01**	**0.39**	**5.54**
	SEM	**0.03**	**0.03**	**0.03**	**0.25**
Males, Day 15	Mean	**0.61**	**1.20**	**0.36**	**6.47**
	SEM	**0.01**	**0.04**	**0.01**	**0.16**
Females, Day 4	Mean	**0.50**	**0.80**	**0.33**	**4.25**
	SEM	**0.03**	**0.03**	**0.04**	**0.23**
Females, Day 15	Mean	**0.51**	**0.84**	**0.31**	**4.49**
	SEM	**0.04**	**0.03**	**0.02**	**0.16**

3.5. Hematology

Total protein was increased in females at Day 15 compared with corresponding baseline values (Figure 3, Table 6 and 7). Remaining parameters were comparable within each sex at baseline, Day 4, and Day 15.

Figure 3.

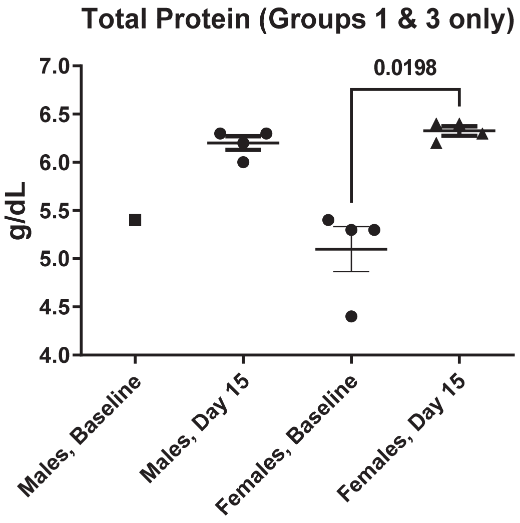

Table 6. Hematology – Baseline Group Means

Group		Total Protein g/dL	RBC x10.e6/uL	Hemoglobin g/dL	Hematocrit %	Hematocrit spun %	MCV fL	MCH pg	MCHC g/dL
Males Day 4 cohort	Mean	5.7	7.0	13.7	40.5	26.0	58.0	19.5	34.0
	SEM	0.1	0.1	0.3	0.5	12.0	1.0	0.5	0.0
Males Day 15 cohort	Mean	5.4	6.4	12.5	39.0	36.0	62.0	20.0	31.0
	SEM	0.0	0.0	0.0	0.0	0.0	0.0	0.0	0.0
Females Day 4 cohort	Mean	5.5	7.1	13.0	37.5	36.5	53.5	18.0	34.5
	SEM	0.7	0.6	1.0	2.5	2.5	0.5	0.0	0.5
Females Day 15 cohort	Mean	5.1	6.6	12.3	36.6	38.3	56.2	19.0	33.2
	SEM	0.2	0.4	0.8	2.5	1.6	1.6	0.3	0.6

Group		RDW %	Platelets x10.e3/uL	MPV fL	WBC x10.e3/uL	Segmented Neutrophils x10.e3/uL	Band Neutrophils x10.e3/uL	Lymphocytes x10.e3/uL	Monocytes x10.e3/uL
Males Day 4 cohort	Mean	12.0	977.0	8.2	11.3	1.1	0.0	49.7	1.0
	SEM	0.0	141.0	0.2	1.5	0.3	0.0	38.3	1.0
Males Day 15 cohort	Mean	12.0	883.0	8.4	12.7	0.5	0.0	11.9	0.3
	SEM	0.0	0.0	0.0	0.0	0.0	0.0	0.0	0.0
Females Day 4 cohort	Mean	10.5	525.0	8.3	7.6	0.9	0.0	6.6	0.1
	SEM	0.5	133.0	0.6	0.2	0.4	0.0	0.5	0.1
Females Day 15 cohort	Mean	10.6	803.8	7.7	8.0	0.7	0.0	7.0	0.2
	SEM	0.4	84.8	0.2	1.3	0.1	0.0	1.3	0.0

Group		Eosinophils x10.e3/uL	Basophils x10.e3/uL	Segmented Neutrophils %	Band Neutrophils %	Lymphocytes %	Monocytes %	Eosinophils %	Basophils %
Males Day 4 cohort	Mean	1.0	0.0	9.5	0.0	88.5	1.0	1.0	0.0
	SEM	1.0	0.0	1.5	0.0	0.5	1.0	1.0	0.0
Males Day 15 cohort	Mean	0.0	0.0	4.0	0.0	94.0	2.0	0.0	0.0
	SEM	0.0	0.0	0.0	0.0	0.0	0.0	0.0	0.0
Females Day 4 cohort	Mean	0.1	0.0	12.0	0.0	86.5	0.5	1.0	0.0
	SEM	0.0	0.0	5.0	0.0	4.5	0.5	0.0	0.0
Females Day 15 cohort	Mean	0.1	0.0	4.0	0.0	86.5	1.7	1.3	0.0
	SEM	0.0	0.0	0.0	0.0	2.2	0.2	0.4	0.0

Table 7. Hematology – Terminal Group Means

Group		Total Protein g/dL	RBC x10.e6/uL	Hemoglobin g/dL	Hematocrit %	Hematocrit spun %	MCV fL	MCH pg	MCHC g/dL
Males, Day 4	Mean	5.7	6.5	12.3	38.2	38.2	59.4	19.0	32.2
	SEM	0.2	0.1	0.2	0.5	0.7	0.5	0.0	0.2
Males, Day 15	Mean	6.2	7.3	14.0	42.5	41.5	58.3	19.0	32.8
	SEM	0.1	0.1	0.2	0.5	0.3	0.3	0.0	0.3
Females, Day 4	Mean	6.0	6.8	12.6	38.0	37.7	56.0	18.7	33.3
	SEM	0.2	0.1	0.2	0.6	1.2	0.0	0.3	0.3
Females, Day 15	Mean	6.3	7.8	14.5	42.8	40.8	55.0	18.8	30.3
	SEM	0.0	0.4	0.7	2.1	2.4	1.1	0.3	3.8

Group		RDW %	Platelets x10.e3/uL	MPV fL	WBC x10.e3/uL	Segmented Neutrophils x10.e3/uL	Band Neutrophils x10.e3/uL	Lymphocytes x10.e3/uL	Monocytes x10.e3/uL
Males, Day 4	Mean	12.4	989.6	8.0	9.2	0.9	0.0	8.0	0.3
	SEM	0.2	117.2	0.1	1.1	0.2	0.0	1.0	0.1
Males, Day 15	Mean	12.0	998.0	8.6	9.4	1.1	0.0	8.0	0.2
	SEM	0.0	60.2	0.2	1.0	0.3	0.0	1.1	0.1
Females, Day 4	Mean	12.0	920.3	8.0	8.5	0.7	0.0	7.5	0.2
	SEM	0.6	18.9	0.2	2.7	0.3	0.0	2.3	0.1
Females, Day 15	Mean	10.8	845.8	8.3	7.9	0.9	0.0	6.7	0.2
	SEM	0.3	60.0	0.5	1.9	0.2	0.0	1.8	0.1

Group		Eosinophils x10.e3/uL	Basophils x10.e3/uL	Segmented Neutrophils %	Band Neutrophils %	Lymphocytes %	Monocytes %	Eosinophils %	Basophils %
Males, Day 4	Mean	0.0	0.0	9.6	0.0	86.6	3.2	0.6	0.0
	SEM	0.0	0.0	2.2	0.0	2.3	0.5	0.2	0.0
Males, Day 15	Mean	0.1	0.0	11.5	0.0	85.3	2.3	1.0	0.0
	SEM	0.1	0.0	3.3	0.0	3.3	0.9	0.7	0.0
Females, Day 4	Mean	0.1	0.0	9.0	0.0	87.3	2.7	1.0	0.0
	SEM	0.1	0.0	2.0	0.0	1.3	0.3	0.6	0.0
Females, Day 15	Mean	0.1	0.0	12.8	0.0	82.8	3.5	1.0	0.0
	SEM	0.0	0.0	2.5	0.0	3.1	0.9	0.4	0.0

3.6. Clinical Biochemistry

Total protein and albumin increased in males from Day 4 to Day 15 (Figure 4, Table 9).

Figure 4.

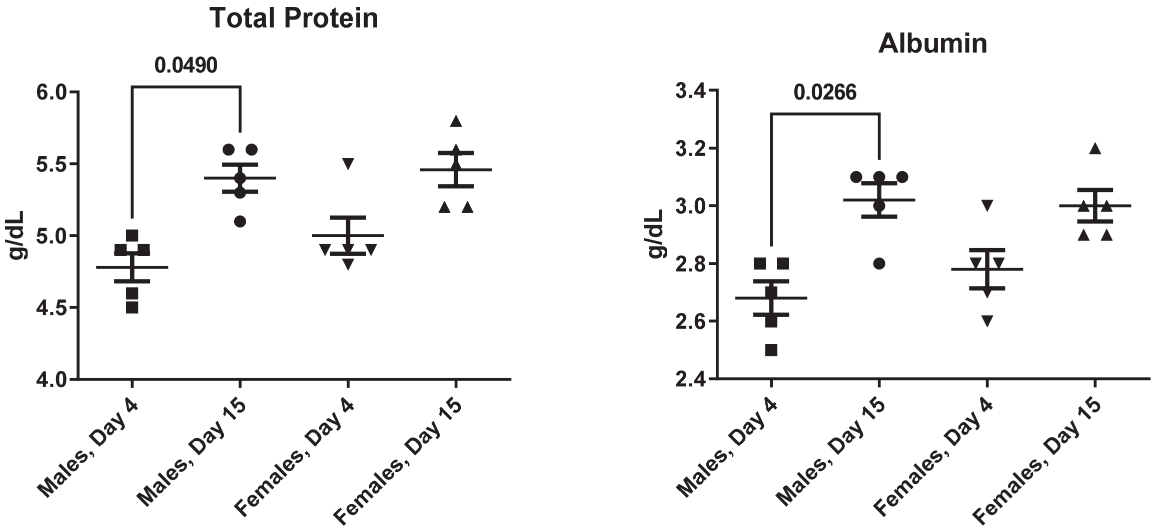

Creatine kinase was decreased in female rats on Days 4 and 15 compared with corresponding baseline levels (Figure 5, Table 8 and 9).

Figure 5.

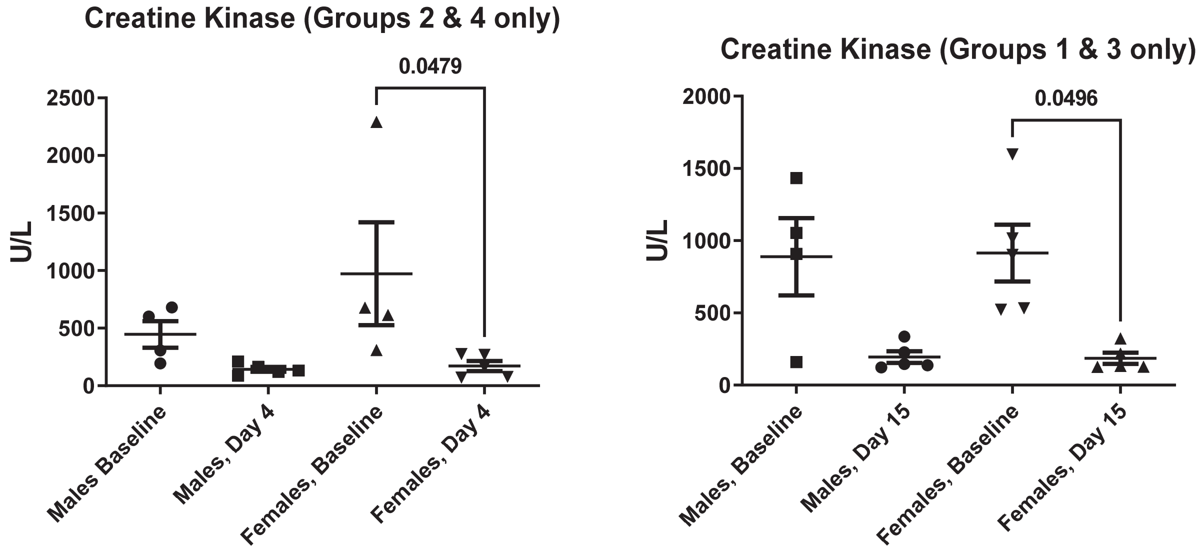

Calcium increased in females from baseline to Day 15 and from Day 4 to Day 15 (Figure 6, Table 8 and 9).

Figure 6.

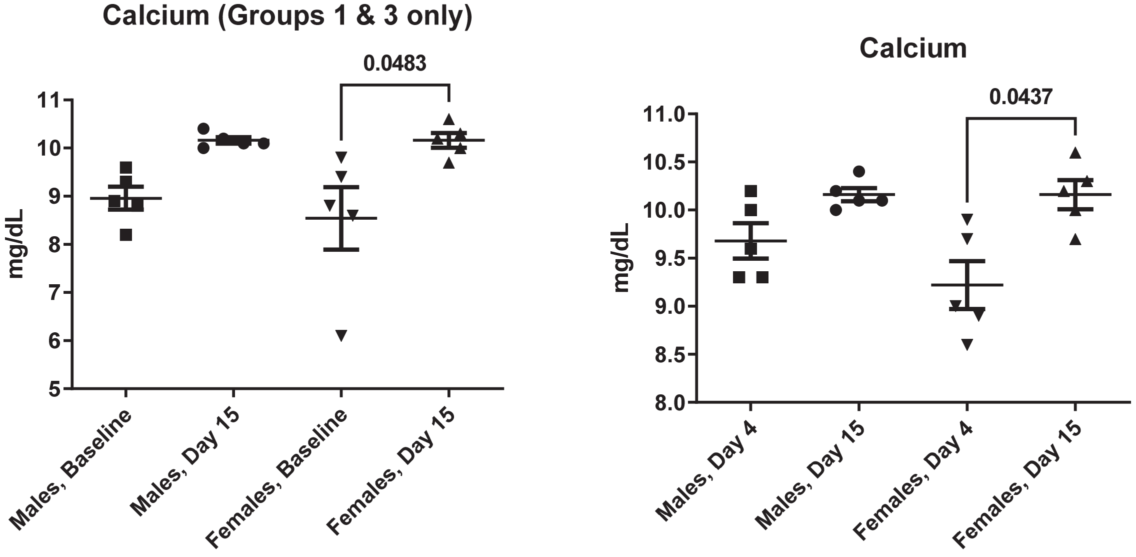

Globulin was increased at Day 15 in females compared with corresponding baseline levels (Figure 7, Table 8 and 9).

Figure 7.

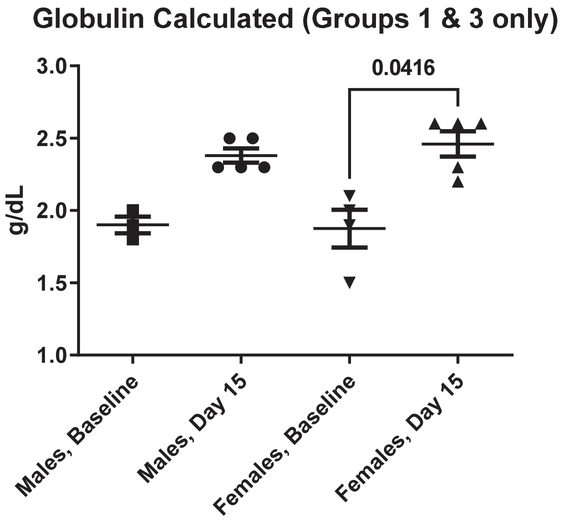

BUN, potassium, and TCO2 increased in females from Day 4 to Day 15 while the Na/K ratio decreased from Day 4 to Day 15 in females (Figure 8, Table 9).

Figure 8.

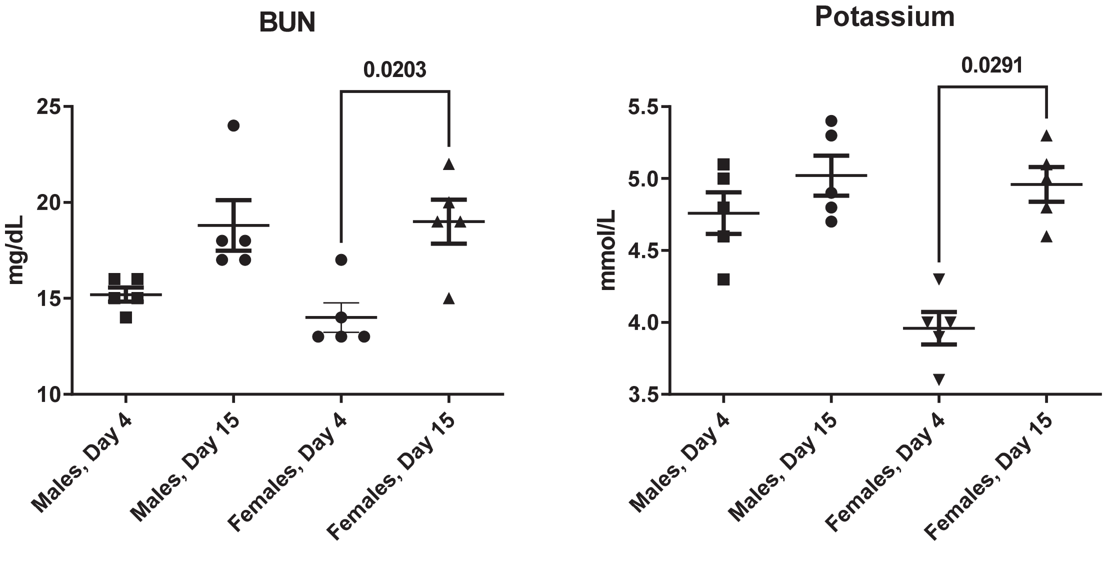

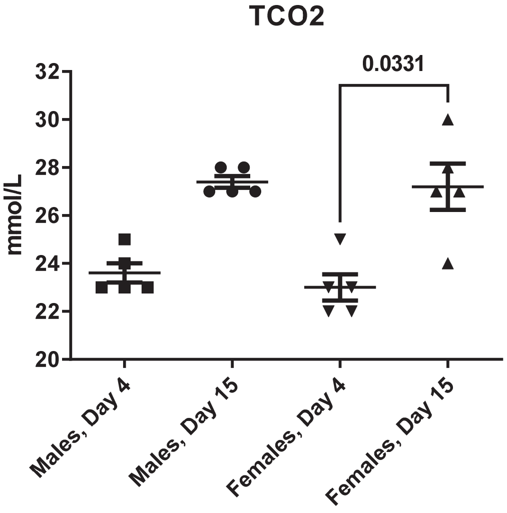

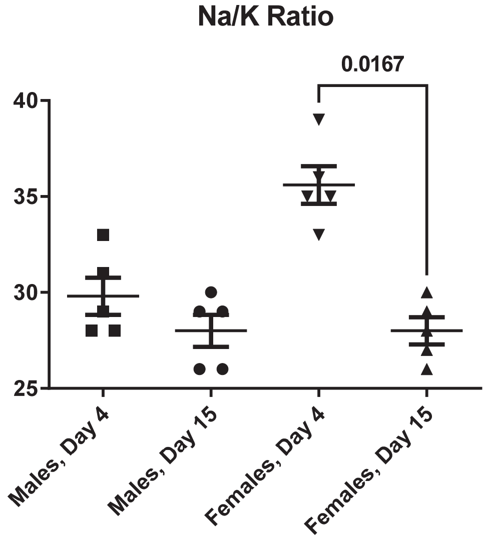

All other biochemistry parameters remained unchanged in males and in females.

Table 8. Biochemistry Parameters – Baseline Group Means

Group		Urea Nitrogen (mg/dL)	Creatinine (Jaffe) (mg/dL)	Sodium (mmol/L)	Potassium (mmol/L)	Chloride (mmol/L)	TCO2 (mmol/L)	Na/K Ratio
Males Day 4 cohort	Mean	**16**	**0.2**	**139**	**5.3**	**102**	**25**	**26**
	SEM	**1**	**0.0**	**1**	**0.2**	**2**	**1**	**1**
Males Day 15 cohort	Mean	**15**	**0.2**	**140**	**5.2**	**104**	**21**	**27**
	SEM	**1**	**0.0**	**1**	**0.4**	**1**	**1**	**2**
Females Day 4 cohort	Mean	**13**	**0.2**	**140**	**4.1**	**109**	**20**	**35**
	SEM	**1**	**0.0**	**1**	**0.2**	**3**	**2**	**2**
Females Day 15 cohort	Mean	**14**	**0.2**	**140**	**4.2**	**107**	**21**	**34**
	SEM	**1**	**0.0**	**1**	**0.4**	**3**	**1**	**4**

Group		Anion Gap (mmol/L)	Osmolarity Calculated (mmol/L)	Calcium (mg/dL)	Phosphorus (mg/dL)	Magnesium (mg/dL)	Iron (ug/dL)	Total Protein (g/dL)
Males Day 4 cohort	Mean	**18**	**293**	**9.5**	**7.1**	**1.7**	**194**	**4.9**
	SEM	**1**	**1**	**0.4**	**0.2**	**0.1**	**13**	**0.2**
Males Day 15 cohort	Mean	**20**	**295**	**9.0**	**8.0**	**1.7**	**225**	**4.5**
	SEM	**1**	**1**	**0.2**	**0.3**	**0.1**	**25**	**0.2**
Females Day 4 cohort	Mean	**16**	**293**	**8.5**	**5.3**	**1.5**	**324**	**5.0**
	SEM	**1**	**1**	**0.7**	**1.0**	**0.2**	**54**	**0.7**
Females Day 15 cohort	Mean	**16**	**294**	**8.5**	**5.6**	**1.5**	**286**	**4.4**
	SEM	**1**	**2**	**0.6**	**0.6**	**0.1**	**9**	**0.3**

Group		Albumin (g/dL)	Globulin Calc (g/dL)	Glucose (Hexokinase) (mg/dL)	Amylase (U/L)	Total Bilirubin (mg/dL)	Direct Bilirubin (mg/dL)	Indirect Bilirubin (mg/dL)
Males Day 4 cohort	Mean	**2.7**	**2.2**	**185**	**461**	**0.1**	**0.0**	**0.1**
	SEM	**0.1**	**0.1**	**16**	**18**	**0.0**	**0.0**	**0.0**
Males Day 15 cohort	Mean	**2.7**	**1.9**	**190**	**457**	**0.1**	**0.0**	**0.1**
	SEM	**0.1**	**0.1**	**7**	**41**	**0.0**	**0.0**	**0.0**
Females Day 4 cohort	Mean	**2.5**	**2.3**	**154**	**279**	**0.1**	**0.0**	**0.1**
	SEM	**0.1**	**0.4**	**18**	**11**	**0.0**	**0.0**	**0.0**
Females Day 15 cohort	Mean	**2.6**	**1.9**	**160**	**274**	**0.1**	**0.0**	**0.1**
	SEM	**0.2**	**0.1**	**14**	**31**	**0.0**	**0.0**	**0.0**

Group		Alkaline Phosphatase (U/L)	Alanine Amino-transferase (U/L)	Aspartate Amino-transferase (U/L)	Creatine Kinase (U/L)	Cholesterol (mg/dL)	Triglyceride (mg/dL)	Lipemia	Icterus	Hemolysis
Males Day 4 cohort	Mean	**385**	**46**	**61**	**446**	**63**	**84**	NA	NA	NA
	SEM	**14**	**3**	**3**	**116**	**5**	**15**	NA	NA	NA
Males Day 15 cohort	Mean	**399**	**40**	**76**	**889**	**65**	**NA**	NA	NA	NA
	SEM	**25**	**3**	**16**	**267**	**3**	**NA**	NA	NA	NA
Females Day 4 cohort	Mean	**171**	**40**	**55**	**973**	**49**	**59**	NA	NA	NA
	SEM	**19**	**3**	**6**	**446**	**3**	**24**	NA	NA	NA
Females Day 15 cohort	Mean	**172**	**40**	**62**	**914**	**48**	**NA**	NA	NA	NA
	SEM	**28**	**6**	**7**	**197**	**4**	**NA**	NA	NA	NA

Table 9. Biochemistry Parameters – Terminal Group Means

Group		Urea Nitrogen (mg/dL)	Creatinine (Jaffe) (mg/dL)	Sodium (mmol/L)	Potassium (mmol/L)	Chloride (mmol/L)	TCO2 (mmol/L)	Na/K Ratio
Males, Day 4	Mean	15	0.2	141	4.8	103	24	30
	SEM	0	0.0	0	0.1	1	0	1
Males, Day 15	Mean	19	0.3	141	5.0	101	27	28
	SEM	1	0.0	0	0.1	0	0	1
Females, Day 4	Mean	14	0.2	141	4.0	106	23	36
	SEM	1	0.0	1	0.1	1	1	1
Females, Day 15	Mean	19	0.2	139	5.0	103	27	28
	SEM	1	0.0	0	0.1	1	1	1

Group		Anion Gap (mmol/L)	Osmolarity Calculated (mmol/L)	Calcium (mg/dL)	Phosphorus (mg/dL)	Magnesium (mg/dL)	Iron (ug/dL)	Total Protein (g/dL)
Males, Day 4	Mean	19	297	9.7	7.3	2.0	267	4.8
	SEM	1	1	0.2	0.3	0.1	34	0.1
Males, Day 15	Mean	17	298	10.2	7.6	2.2	220	5.4
	SEM	1	1	0.1	0.0	0.0	11	0.1
Females, Day 4	Mean	16	296	9.2	6.1	1.9	303	5.0
	SEM	0	1	0.2	0.4	0.1	20	0.1
Females, Day 15	Mean	14	294	10.2	6.9	2.0	304	5.5
	SEM	0	0	0.2	0.3	0.1	25	0.1

Group		Albumin (g/dL)	Globulin Calc (g/dL)	Glucose (Hexokinase) (mg/dL)	Amylase (U/L)	Total Bilirubin (mg/dL)	Direct Bilirubin (mg/dL)	Indirect Bilirubin (mg/dL)
Males, Day 4	Mean	2.7	2.1	157	459	0.1	0.0	0.1
	SEM	0.1	0.0	4	15	0.0	0.0	0.0
Males, Day 15	Mean	3.0	2.4	176	543	0.1	0.0	0.1
	SEM	0.1	0.1	9	21	0.0	0.0	0.0
Females, Day 4	Mean	2.8	2.2	163	296	0.1	0.0	0.1
	SEM	0.1	0.1	10	10	0.0	0.0	0.0
Females, Day 15	Mean	3.0	2.5	160	332	0.1	0.0	0.1
	SEM	0.1	0.1	4	13	0.0	0.0	0.0

Group		Alkaline Phosphatase (U/L)	Alanine Amino-transferase (U/L)	Aspartate Amino-transferase (U/L)	Creatine Kinase (U/L)	Cholesterol (mg/dL)	Triglyceride (mg/dL)	Lipemia	Icterus	Hemolysis
Males, Day 4	Mean	365	46	55	143	60	75	NA	NA	NA
	SEM	18	3	2	21	3	7	NA	NA	NA
Males, Day 15	Mean	380	46	65	194	64	81	NA	NA	NA
	SEM	26	2	5	40	2	9	NA	NA	NA
Females, Day 4	Mean	164	39	44	172	51	46	NA	NA	NA
	SEM	23	4	2	44	2	4	NA	NA	NA
Females, Day 15	Mean	199	50	62	185	58	NA	NA	NA	NA
	SEM	10	3	6	38	2	NA	NA	NA	NA

3.7. Histopathology

There was little to no variation between animals within or between treatment groups with the exception of the brain (Appendix V). Mild vacuolation of the white matter was observed throughout the brain at 4 days post dose. There were no indications of this lesion at Day 15, however. Epicardial hemorrhage was noted in one animal and minimal renal cortical mineralization noted in another; both of these latter lesions were considered to be background and not significant to the study.

4. CONCLUSION

UM_COU was clinically well-tolerated when administered as a single intravenous bolus to male and female adult Sprague-Dawley rats, evaluated through 15 days after dosing. There were no adverse effects on body weight or food consumption. Biochemistry changes were minor and limited to increases in total protein and albumin in males, decreased creatinine kinase levels and Na/K ratios in females, and increased calcium, globulin, BUN, potassium, and TCO2 levels in females. Mild vacuolation of brain white matter was observed microscopically at 4 days post dose. However, there were no indications of this lesion by 15 days post dose.

Appendix I. Individual Body Weight over Time

Body Weight (g)

Group	Animal No.	Study Day		
		1	4	15
1 Males Day 15 cohort	101	201.7	NA	306.1
	102	189.3	NA	268.3
	103	196.2	NA	286.3
	104	191.8	NA	290.6
	105	183.7	NA	273.1
2 Males Day 4 cohort	201	227.1	248.7	NA
	202	225.6	241.7	NA
	203	232.0	251.7	NA
	204	228.6	247.1	NA
	205	240.5	262.2	NA
3 Females Day 15 cohort	301	204.1	NA	227.5
	302	207.5	NA	232.0
	303	229	NA	260.4
	304	202.6	NA	229.6
	305	199.8	NA	216.8
4 Females Day 4 cohort	401	217.8	218.4	NA
	402	224.5	221.2	NA
	403	224.6	227.6	NA
	404	218.8	222.5	NA
	405	215.3	218.3	NA

Appendix II. Individual Organ Weights

Organ Weight (g)						
Group	Animal No.	Brain	Heart	Liver	Spleen	Kidneys (L&R combined)
Males, Day 4	201	**1.669**	**0.902**	**9.953**	**0.675**	**1.629**
	202	**1.651**	**1.026**	**8.694**	**0.522**	**1.751**
	203	**1.653**	**0.873**	**10.054**	**0.791**	**1.759**
	204	**1.881**	**0.949**	**8.816**	**0.676**	**1.750**
	205	**1.813**	**1.165**	**10.333**	**0.740**	**1.871**
Males, Day 15	101	**1.771**	**1.121**	**12.284**	**0.568**	**2.228**
	102	**1.735**	**1.027**	**10.592**	**0.654**	**1.814**
	103	**1.775**	**1.025**	**11.613**	**0.651**	**2.147**
	104	**1.670**	**1.069**	**11.127**	**0.633**	**2.094**
	105	**1.736**	**1.054**	**10.622**	**0.637**	**2.163**
Females, Day 4	401	**1.606**	**1.034**	**8.084**	**0.734**	**1.363**
	402	**1.818**	**0.885**	**7.497**	**0.598**	**1.479**
	403	**1.934**	**0.897**	**8.455**	**0.649**	**1.475**
	404	**2.012**	**0.927**	**7.269**	**0.571**	**1.436**
	405	**1.875**	**0.887**	**7.703**	**0.467**	**1.600**
Females, Day 15	301	**1.825**	**0.814**	**8.003**	**0.652**	**1.395**
	302	**1.713**	**0.904**	**8.272**	**0.538**	**1.590**
	303	**1.848**	**1.259**	**8.991**	**0.615**	**1.590**
	304	**1.732**	**0.782**	**7.522**	**0.495**	**1.310**
	305	**1.829**	**0.842**	**7.337**	**0.460**	**1.597**

Organ Weight:Body Weight						
**Group**	**Animal No.**	**Brain:BW Ratio**	**Heart:BW Ratio**	**Spleen:BW Ratio**	**Kidneys:BW Ratio**	**Liver:BW Ratio**
Males, Day 4	201	**0.0067**	**0.0036**	**0.0027**	**0.0065**	**0.0400**
	202	**0.0068**	**0.0042**	**0.0022**	**0.0072**	**0.0360**
	203	**0.0066**	**0.0035**	**0.0031**	**0.0070**	**0.0399**
	204	**0.0076**	**0.0038**	**0.0027**	**0.0071**	**0.0357**
	205	**0.0069**	**0.0044**	**0.0028**	**0.0071**	**0.0394**
Males, Day 15	101	**0.0058**	**0.0037**	**0.0019**	**0.0073**	**0.0401**
	102	**0.0065**	**0.0038**	**0.0024**	**0.0068**	**0.0395**
	103	**0.0062**	**0.0036**	**0.0023**	**0.0075**	**0.0406**
	104	**0.0057**	**0.0037**	**0.0022**	**0.0072**	**0.0383**
	105	**0.0064**	**0.0039**	**0.0023**	**0.0079**	**0.0389**
Females, Day 4	401	**0.0074**	**0.0047**	**0.0034**	**0.0062**	**0.0370**
	402	**0.0082**	**0.0040**	**0.0027**	**0.0067**	**0.0339**
	403	**0.0085**	**0.0039**	**0.0029**	**0.0065**	**0.0371**
	404	**0.0090**	**0.0042**	**0.0026**	**0.0065**	**0.0327**
	405	**0.0086**	**0.0041**	**0.0021**	**0.0073**	**0.0353**
Females, Day 15	301	**0.0080**	**0.0036**	**0.0029**	**0.0061**	**0.0352**
	302	**0.0074**	**0.0039**	**0.0023**	**0.0069**	**0.0356**
	303	**0.0071**	**0.0048**	**0.0024**	**0.0061**	**0.0345**
	304	**0.0075**	**0.0034**	**0.0022**	**0.0057**	**0.0328**
	305	**0.0084**	**0.0039**	**0.0021**	**0.0074**	**0.0339**

Organ Weight:Brain Weight					
**Group**	**Animal No.**	**Heart:Brain Wt Ratio**	**Spleen:Brain Wt Ratio**	**Kidneys:Brain Wt Ratio**	**Liver:Brain Wt Ratio**
Males, Day 4	201	**0.54**	**0.40**	**0.98**	**5.96**
	202	**0.62**	**0.32**	**1.06**	**5.27**
	203	**0.53**	**0.48**	**1.06**	**6.08**
	204	**0.50**	**0.36**	**0.93**	**4.69**
	205	**0.64**	**0.41**	**1.03**	**5.70**
Males, Day 15	101	**0.63**	**0.32**	**1.26**	**6.94**
	102	**0.59**	**0.38**	**1.05**	**6.10**
	103	**0.58**	**0.37**	**1.21**	**6.54**
	104	**0.64**	**0.38**	**1.25**	**6.66**
	105	**0.61**	**0.37**	**1.25**	**6.12**
Females, Day 4	401	**0.64**	**0.46**	**0.85**	**5.03**
	402	**0.49**	**0.33**	**0.81**	**4.12**
	403	**0.46**	**0.34**	**0.76**	**4.37**
	404	**0.46**	**0.28**	**0.71**	**3.61**
	405	**0.47**	**0.25**	**0.85**	**4.11**
Females, Day 15	301	**0.45**	**0.36**	**0.76**	**4.39**
	302	**0.53**	**0.31**	**0.93**	**4.83**
	303	**0.68**	**0.33**	**0.86**	**4.87**
	304	**0.45**	**0.29**	**0.76**	**4.34**
	305	0.46	0.25	0.87	4.01

Appendix III. Individual Hematology Values

BASELINE VALUES

Group	Animal No.	Total Protein g/dL	RBC x10.e6/uL	Hemoglobin g/dL	Hematocrit %	Hematocrit spun %	MCV fL	MCH pg	MCHC g/dL
1 Male Day 15 cohort	101	NA	NA	NA	NA	NA	NA	NA	NA
	102	NA	NA	NA	NA	NA	NA	NA	NA
	103	NA	NA	NA	NA	NA	NA	NA	NA
	104	NA	NA	NA	NA	NA	NA	NA	NA
	105	5.4	6.4	12.5	39	36	62	20	31
2 Male Day 4 cohort	201	5.6	6.9	14	41	14	59	20	34
	202	NA	NA	NA	NA	NA	NA	NA	NA
	203	NA	NA	NA	NA	NA	NA	NA	NA
	204	NA	NA	NA	NA	NA	NA	NA	NA
	205	5.7	7.0	13.4	40	38	57	19	34
3 Female Day 15 cohort	301	5.3	7.3	13.7	40.0	41.0	55.0	19.0	34.0
	302	5.3	7.3	13.8	41.0	41.0	56.0	19.0	33.0
	303	5.4	6.4	12.5	39	36	62	20	31
	304	QNS	4.9	9.3	27	QNS	56	19	34
	305	4.4	6.9	12.4	36	35	52	18	34
4 Female Day 4 cohort	401	4.8	6.5	12	35	34	54	18	34
	402	NA	NA	NA	NA	NA	NA	NA	NA
	403	NA	NA	NA	NA	NA	NA	NA	NA
	404	NA	NA	NA	NA	NA	NA	NA	NA
	405	6.1	7.6	14.0	40	39	53	18	35

Group	Animal No.	RDW %	Platelets x10.e3/uL	MPV fL	WBC x10.e3/uL	Segmented Neutrophils x10.e3/uL	Band Neutrophils x10.e3/uL	Lymphocytes x10.e3/uL	Monocytes x10.e3/uL
1 Male Day 15 cohort	101	NA	NA	NA	NA	NA	NA	NA	NA
	102	NA	NA	NA	NA	NA	NA	NA	NA
	103	NA	NA	NA	NA	NA	NA	NA	NA
	104	NA	NA	NA	NA	NA	NA	NA	NA
	105	12.0	883.0	8.4	13	1	0	12	0
2 Male Day 4 cohort	201	12	836	8.3	9.8	0.8	0.0	88	2
	202	NA	NA	NA	NA	NA	NA	NA	NA
	203	NA	NA	NA	NA	NA	NA	NA	NA
	204	NA	NA	NA	NA	NA	NA	NA	NA
	205	12.0	1118.0	8.0	13	1	0	11	0
3 Female Day 15 cohort	301	11.0	1075.0	7.5	8.4	0.7	NA	7.4	0.2
	302	10.0	752.0	7.5	6.6	0.8	NA	5.6	0.1
	303	12.0	883.0	8.4	13	1	0	12	0
	304	10.0	563.0	7.6	7	1	NA	6	0
	305	10.0	746.0	7.6	6	1	NA	5	0
4 Female Day 4 cohort	401	10	658	7.7	7.4	1.3	0.0	6.1	0.0
	402	NA	NA	NA	NA	NA	NA	NA	NA
	403	NA	NA	NA	NA	NA	NA	NA	NA
	404	NA	NA	NA	NA	NA	NA	NA	NA
	405	11.0	392.0	8.8	8	1	0	7	0

BASELINE VALUES, cont.

Group	Animal No.	Eosinophils x10.e3/uL	Basophils x10.e3/uL	Segmented Neutrophils %	Band Neutrophils %	Lymphocytes %	Monocytes %	Eosinophils %	Basophils %
1 Male Day 15 cohort	101	NA	NA	NA	NA	NA	NA	NA	NA
	102	NA	NA	NA	NA	NA	NA	NA	NA
	103	NA	NA	NA	NA	NA	NA	NA	NA
	104	NA	NA	NA	NA	NA	NA	NA	NA
	105	0.0	0.0	4.0	0	94	2	0	0
2 Male Day 4 cohort	201	2	0.0	8	0	88	2	2	0
	202	NA	NA	NA	NA	NA	NA	NA	NA
	203	NA	NA	NA	NA	NA	NA	NA	NA
	204	NA	NA	NA	NA	NA	NA	NA	NA
	205	0.0	0.0	11.0	0	89	0	0	0
3 Female Day 15 cohort	301	0.1	NA	NA	7.8	87.6	2.2	1.5	NA
	302	0.1	NA	NA	12.0	84.3	1.4	1.8	NA
	303	0.0	NA	4.0	0	94	2	0	0
	304	0.1	NA	NA	11	86	1	1	NA
	305	0.1	NA	NA	15	81	2	2	NA
4 Female Day 4 cohort	401	0.1	0.0	17	0	82	0	1	0
	402	NA	NA	NA	NA	NA	NA	NA	NA
	403	NA	NA	NA	NA	NA	NA	NA	NA
	404	NA	NA	NA	NA	NA	NA	NA	NA
	405	0.1	0.0	7.0	0	91	1	1	0

Abbreviations:

MCV = Mean Cell Volume
MCH = Mean Corpuscular Hemoglobin
MCHC = Mean Cell Hemoglobin Concentration
RDW = Red Cell Distribution Width
MPV = Mean Platelet Volume
WBC = White Blood Cells

TERMINAL VALUES

Group	Animal No.	Total Protein g/dL	RBC x10.e6/uL	Hemoglobin g/dL	Hematocrit %	Hematocrit spun %	MCV fL	MCH pg	MCHC g/dL
1 Male Day 15 cohort	101	6.3	7.2	13.8	42.0	41.0	59.0	19.0	32.0
	102	6.2	7.6	14.6	44.0	42.0	58.0	19.0	33.0
	103	6.3	7.2	13.8	42	41	58	19	33
	104	6.0	7.3	13.8	42	42	58	19	33
	105	NA	NA	NA	NA	NA	NA	NA	NA
2 Male Day 4 cohort	201	5.8	6.5	12.4	39	40	60	19	32
	202	5.3	6.5	12.6	39	37	61	19	32
	203	6.2	6.4	12.0	37.0	40.0	58.0	19.0	32.0
	204	5.6	6.7	12.7	39	37	59	19	33
	205	5.5	6.3	11.8	37	37	59	19	32
3 Female Day 15 cohort	301	NA	NA	NA	NA	NA	NA	NA	NA
	302	6.4	6.6	12.4	37.0	34.0	56.0	19.0	34.0
	303	6.3	7.8	14.7	42	41	55	19	19
	304	6.4	8.1	15.3	46	44	57	19	34
	305	6.2	8.7	15.4	46	44	52	18	34
4 Female Day 4 cohort	401	6	6.8	12.5	38	37	56	18	33
	402	6.3	6.9	12.9	39	40	56	19	33
	403	5.6	6.6	12.4	37.0	36.0	56.0	19.0	34.0
	404	NA	NA	NA	NA	NA	NA	NA	NA
	405	NA	NA	NA	NA	NA	NA	NA	NA

Group	Animal No.	RDW %	Platelets x10.e3/uL	MPV fL	WBC x10.e3/uL	Segmented Neutrophils x10.e3/uL	Band Neutrophils x10.e3/uL	Lymphocytes x10.e3/uL	Monocytes x10.e3/uL
1 Male Day 15 cohort	101	12.0	1169.0	8.3	8.0	0.9	0.0	6.8	0.3
	102	12.0	915.0	8.1	7.5	1.0	0.0	6.3	0.2
	103	12.0	996.0	9.1	12	0	0	11	0
	104	12.0	912.0	8.8	10	2	0	8	0
	105	NA	NA	NA	NA	NA	NA	NA	NA
2 Male Day 4 cohort	201	12	1087	7.6	10.8	0.6	0	9.8	0.3
	202	13.0	1077.0	8.0	7	1	0	6	0
	203	12.0	1032.0	8.0	8.4	1.4	0.0	6.6	0.3
	204	12.0	537.0	8.3	7	0	0	7	0
	205	13.0	1215.0	7.9	13	1	0	11	1
3 Female Day 15 cohort	301	NA	NA	NA	NA	NA	NA	NA	NA
	302	11.0	784.0	7.9	2.5	0.4	0.0	1.9	0.1
	303	11.0	726.0	9.9	9	1	0	7	0
	304	11.0	1002.0	8.0	12	1	0	10	0
	305	10.0	871.0	7.5	9	1	0	8	0
4 Female Day 4 cohort	401	13	890	8.1	12.9	1.4	0	11.1	0.3
	402	12.0	916.0	7.7	4	0	0	3	0
	403	11.0	955.0	8.2	9.0	0.4	0.0	8.1	0.3
	404	NA	NA	NA	NA	NA	NA	NA	NA
	405	NA	NA	NA	NA	NA	NA	NA	NA

TERMINAL VALUES, cont.

Group	Animal No.	Eosinophils x10.e3/uL	Basophils x10.e3/uL	Segmented Neutrophils %	Band Neutrophils %	Lymphocytes %	Monocytes %	Eosinophils %	Basophils %
1 Male Day 15 cohort	101	0.0	0.0	11.0	0.0	85.0	4.0	0.0	0.0
	102	0.1	0.0	13.0	0.0	84.0	2.0	1.0	0.0
	103	0.4	0.0	3.0	0	94	0	3	0
	104	0.0	0.0	19.0	0	78	3	0	0
	105	NA	NA	NA	NA	NA	NA	NA	NA
2 Male Day 4 cohort	201	0	0	6	0	91	3	0	0
	202	0.1	0.0	12.0	0	85	2	1	0
	203	0.0	0.0	17.0	0.0	79.0	3.0	1.0	0.0
	204	0.0	0.0	5.0	0	92	3	0	0
	205	0.1	0.0	8.0	0	86	5	1	0
3 Female Day 15 cohort	301	NA	NA	NA	NA	NA	NA	NA	NA
	302	0.0	0.0	18.0	0.0	77.0	5.0	0.0	0.0
	303	0.1	0.0	16.0	0	78	5	1	0
	304	0.1	0.0	8.0	0	89	2	1	0
	305	0.2	0.0	9.0	0	87	2	2	0
4 Female Day 4 cohort	401	0.1	0	11	0	86	2	1	0
	402	0.0	0.0	11.0	0	86	3	0	0
	403	0.2	0.0	5.0	0.0	90.0	3.0	2.0	0.0
	404	NA	NA	NA	NA	NA	NA	NA	NA
	405	NA	NA	NA	NA	NA	NA	NA	NA

Abbreviations:

MCV = Mean Cell Volume
MCH = Mean Corpuscular Hemoglobin
MCHC = Mean Cell Hemoglobin Concentration
RDW = Red Cell Distribution Width
MPV = Mean Platelet Volume
WBC = White Blood Cells

Appendix IV. Individual Biochemistry Values

BASELINE VALUES

Group	Animal No.	Urea Nitrogen (mg/dL)	Creatinine (Jaffe) (mg/dL)	Sodium (mmol/L)	Potassium (mmol/L)	Chloride (mmol/L)	TCO2 (mmol/L)	Na/K Ratio
1 (Male Day 15)	101	13	QNS	139	5.8	QNS	QNS	24
	102	20	0.2	140	6.3	102	22	22
	103	14	0.2	141	4.4	104	22	32
	104	16	QNS	138	5.3	QNS	QNS	26
	105	13	0.2	140	4.3	105	20	33
2 (Male Day 4)	201	19	0.2	137	6.0	99	24	23
	202	15	0.2	138	5.2	102	QNS	27
	203	16	0.2	137	5.6	100	25	24
	204	17	0.2	139	5.1	101	26	27
	205	15	0.2	142	4.8	109	23	30
3 (Female Day 15)	301	18	0.2	137	5.4	102	23	25
	302	11	0.2	138	4.7	102	24	29
	303	11	0.2	140	4.0	107	22	35
	304	14	<0.1	145	2.9	119	16	50
	305	14	0.2	140	4.2	107	21	33
4 (Female Day 4)	401	15	0.2	140	3.7	112	19	38
	402	14	0.2	141	4.7	112	16	30
	403	13	0.2	139	3.9	109	18	36
	404	16	0.2	138	4.5	98	27	31
	405	9	QNS	141	3.5	112	QNS	40

BASELINE VALUES, cont.

Group	Animal No.	Anion Gap (mmol/L)	Osmolarity Calculated (mmol/L)	Calcium (mg/dL)	Phosphorus (mg/dL)	Magnesium (mg/dL)	Iron (ug/dL)	Total Protein (g/dL)
1 (Male Day 15)	101	QNS	292	9.3	8.4	1.9	QNS	QNS
	102	22	298	9.6	8.8	1.8	190	4.8
	103	19	298	8.8	7.5	1.7	213	4.5
	104	QNS	293	8.9	8.2	1.8	QNS	QNS
	105	19	294	8.2	7.3	1.5	273	4.2
2 (Male Day 4)	201	20	290	10.6	7.5	1.8	229	5.3
	202	QNS	294	8.2	7.0	1.6	QNS	QNS
	203	18	291	10.0	7.3	1.7	197	5.0
	204	17	295	10.0	7.4	1.8	169	4.9
	205	15	297	8.8	6.2	1.5	180	4.3
3 (Female Day 15)	301	17	291	9.4	6.8	1.8	291	4.7
	302	17	290	9.8	QNS	1.5	QNS	QNS
	303	15	293	8.6	5.8	1.6	306	4.5
	304	13	302	6.1	3.8	1.1	264	3.4
	305	16	292	8.8	6.0	1.3	281	4.8
4 (Female Day 4)	401	13	293	7.7	4.3	1.5	286	4.3
	402	18	294	7.4	QNS	1.2	256	QNS
	403	16	291	8.4	QNS	QNS	QNS	QNS
	404	18	293	10.5	6.3	1.9	430	5.6
	405	QNS	QNS	QNS	QNS	QNS	QNS	QNS

BASELINE VALUES, cont.

Group	Animal No.	Albumin (g/dL)	Globulin Calc (g/dL)	Glucose (Hexokinase) (mg/dL)	Amylase (U/L)	Total Bilirubin (mg/dL)	Direct Bilirubin (mg/dL)	Indirect Bilirubin (mg/dL)
1 (Male Day 15)	101	3.0	QNS	176	QNS	0.2	QNS	QNS
	102	2.8	2.0	187	476	0.1	0.0	0.1
	103	2.6	1.9	200	516	0.1	0.0	0.1
	104	QNS	QNS	212	QNS	0.2	QNS	QNS
	105	2.4	1.8	176	378	0.1	0.0	0.1
2 (Male Day 4)	201	2.8	2.5	171	509	0.1	0.0	0.1
	202	2.6	QNS	222	446	0.1	QNS	QNS
	203	2.7	2.3	211	469	0.1	0.0	0.1
	204	2.8	2.1	188	479	0.1	0.0	0.1
	205	2.4	1.9	132	400	0.1	0.0	0.1
3 (Female Day 15)	301	2.6	2.1	186	362	0.1	0.0	0.1
	302	2.9	QNS	190	281	QNS	0.0	QNS
	303	2.6	1.9	172	258	0.1	0.0	0.1
	304	1.9	1.5	120	173	0.1	0.0	0.1
	305	2.8	2.0	132	296	0.1	0.0	0.1
4 (Female Day 4)	401	2.4	1.9	146	271	0.1	0.0	0.1
	402	2.5	QNS	120	261	0.1	0.0	0.1
	403	2.4	QNS	146	285	0.1	QNS	QNS
	404	3.0	2.6	204	320	0.1	0.0	0.1
	405	2.3	QNS	QNS	256	QNS	QNS	QNS

BASELINE VALUES, cont.

Group	Animal No.	Alkaline Phosphatase (U/L)	Alanine Amino-transferase (U/L)	Aspartate Amino-transferase (U/L)	Creatine Kinase (U/L)	Cholesterol (mg/dL)	Triglyceride (mg/dL)	Lipemia	Icterus	Hemolysis
1 (Male Day 15)	101	439	45	70	1054	67	NA	Normal	Normal	Normal
	102	338	35	52	159	64	NA	Normal	Normal	Normal
	103	404	34	57	909	67	NA	Normal	Normal	Normal
	104	QNS	51	139	QNS	73	NA	Normal	Normal	Normal
	105	414	37	64	1432	55	NA	Normal	Normal	Normal
2 (Male Day 4)	201	354	50	67	680	82	127	Normal	Normal	Normal
	202	408	41	60	QNS	56	NA	Slight	Normal	Normal
	203	400	47	62	602	65	88	Normal	Normal	Normal
	204	415	52	63	308	58	50	Normal	Normal	Normal
	205	347	39	51	194	53	69	Normal	Normal	Normal
3 (Female Day 15)	301	231	60	80	902	50	NA	Slight	Normal	Normal
	302	227	40	78	1597	49	NA	Normal	Normal	Normal
	303	138	35	54	1015	57	NA	Slight	Normal	Normal
	304	84	27	46	523	34	NA	Slight	Normal	Normal
	305	181	36	51	533	50	NA	Normal	Normal	Normal
4 (Female Day 4)	401	216	53	76	2290	41	35	Slight	Normal	Normal
	402	191	36	53	678	48	21	Slight	Normal	Normal
	403	196	36	54	613	54	NA	Normal	Normal	Normal
	404	133	39	51	310	51	122	Slight	Normal	Normal
	405	119	37	39	QNS	QNS	NA	Slight	Normal	Normal

TERMINAL VALUES

Group	Animal No.	Urea Nitrogen (mg/dL)	Creatinine (Jaffe) (mg/dL)	Sodium (mmol/L)	Potassium (mmol/L)	Chloride (mmol/L)	TCO2 (mmol/L)	Na/K Ratio
1 (Male Day 15)	101	18	0.3	140	5.3	100	28	26
	102	24	0.3	141	5.4	101	27	26
	103	18	0.2	142	4.7	101	27	30
	104	17	0.3	140	4.8	101	28	29
	105	17	0.2	141	4.9	102	27	29
2 (Male Day 4)	201	16	0.3	140	4.8	101	24	29
	202	15	0.2	142	5.1	103	23	28
	203	14	0.2	142	4.3	105	23	33
	204	16	0.2	140	5.0	101	25	28
	205	15	0.2	142	4.6	106	23	31
3 (Female Day 15)	301	22	0.2	139	5.3	102	27	26
	302	15	0.2	140	4.6	106	24	30
	303	19	0.3	139	5.1	100	30	27
	304	20	0.3	139	5.0	103	27	28
	305	19	0.2	139	4.8	102	28	29
4 (Female Day 4)	401	13	0.2	141	4.3	108	22	33
	402	13	0.3	141	4.0	104	25	35
	403	17	0.2	138	3.9	103	23	35
	404	14	0.3	142	3.6	108	22	39
	405	13	0.2	142	4.0	107	23	36

TERMINAL VALUES, cont.

Group	Animal No.	Anion Gap (mmol/L)	Osmolarity Calculated (mmol/L)	Calcium (mg/dL)	Phosphorus (mg/dL)	Magnesium (mg/dL)	Iron (ug/dL)	Total Protein (g/dL)
1 (Male Day 15)	101	17	297	10.4	7.5	2.3	226	5.6
	102	18	300	10.2	7.7	2.1	197	5.4
	103	19	300	10.1	7.7	2.3	200	5.6
	104	16	298	10.0	7.6	2.1	231	5.1
	105	17	297	10.1	7.6	2.1	247	5.3
2 (Male Day 4)	201	20	294	10.2	7.9	2.1	306	4.9
	202	21	298	9.6	7.9	1.9	384	5.0
	203	18	298	9.3	6.5	1.8	211	4.6
	204	19	295	10.0	7.1	1.9	226	4.9
	205	18	298	9.3	7.3	2.2	209	4.5
3 (Female Day 15)	301	15	294	10.0	7.5	2.0	289	5.5
	302	15	294	9.7	5.7	1.8	320	5.2
	303	14	293	10.6	7.2	2.2	393	5.8
	304	14	295	10.2	7.0	2.1	255	5.6
	305	14	294	10.3	7.1	1.9	264	5.2
4 (Female Day 4)	401	15	295	9.0	5.5	1.7	277	4.9
	402	16	296	9.7	5.7	2.2	241	5.5
	403	16	293	9.9	7.4	2.0	332	4.9
	404	16	297	8.6	5.6	1.8	306	4.9
	405	16	298	8.9	6.2	1.7	358	4.8

TERMINAL VALUES, cont.

Group	Animal No.	Albumin (g/dL)	Globulin Calc (g/dL)	Glucose (Hexokinase) (mg/dL)	Amylase (U/L)	Total Bilirubin (mg/dL)	Direct Bilirubin (mg/dL)	Indirect Bilirubin (mg/dL)
1 (Male Day 15)	101	3.1	2.5	182	584	0.1	0.0	0.1
	102	3.1	2.3	162	515	0.1	0.0	0.1
	103	3.1	2.5	172	603	0.1	0.0	0.1
	104	2.8	2.3	207	491	0.1	0.0	0.1
	105	3.0	2.3	155	522	0.1	0.0	0.1
2 (Male Day 4)	201	2.7	2.2	153	472	0.1	0.0	0.1
	202	2.8	2.2	151	476	0.1	0.0	0.1
	203	2.6	2.0	157	449	0.1	0.0	0.1
	204	2.8	2.1	174	493	0.1	0.0	0.1
	205	2.5	2.0	150	407	0.1	0.0	0.1
3 (Female Day 15)	301	2.9	2.6	153	357	0.1	0.0	0.1
	302	2.9	2.3	161	292	0.1	0.0	0.1
	303	3.2	2.6	149	362	0.2	0.0	0.2
	304	3.0	2.6	175	322	0.1	0.0	0.1
	305	3.0	2.2	160	325	0.1	0.0	0.1
4 (Female Day 4)	401	2.8	2.1	144	284	0.1	0.0	0.1
	402	3.0	2.5	174	300	0.1	0.0	0.1
	403	2.7	2.2	197	312	0.1	0.0	0.1
	404	2.8	2.1	140	263	0.1	0.0	0.1
	405	2.6	2.2	161	322	0.1	0.0	0.1

TERMINAL VALUES, cont.

Group	Animal No.	Alkaline Phosphatase (U/L)	Alanine Amino-transferase (U/L)	Aspartate Amino-transferase (U/L)	Creatine Kinase (U/L)	Cholesterol (mg/dL)	Triglyceride (mg/dL)	Lipemia	Icterus	Hemolysis
1 (Male Day 15)	101	383	52	66	228	62	103	Normal	Normal	Normal
	102	322	43	81	336	61	97	Normal	Normal	Normal
	103	400	42	63	123	64	63	Normal	Normal	Normal
	104	332	43	53	145	71	86	Normal	Normal	Normal
	105	464	51	61	138	63	57	Normal	Normal	Normal
2 (Male Day 4)	201	353	51	60	166	72	70	Normal	Normal	Normal
	202	393	43	56	133	59	100	Normal	Normal	Normal
	203	313	42	48	119	56	74	Normal	Normal	Normal
	204	417	54	59	211	56	54	Normal	Normal	Normal
	205	347	38	51	87	56	76	Normal	Normal	Normal
3 (Female Day 15)	301	189	57	80	217	54	NA	Normal	Normal	Normal
	302	205	49	51	128	56	NA	Normal	Normal	Normal
	303	233	54	68	323	63	NA	Normal	Normal	Normal
	304	171	49	60	125	63	NA	Normal	Normal	Normal
	305	198	42	50	133	52	NA	Normal	Normal	Normal
4 (Female Day 4)	401	170	50	48	78	49	45	Normal	Normal	Normal
	402	226	43	42	71	59	29	Normal	Normal	Normal
	403	193	40	50	276	53	51	Normal	Normal	Normal
	404	87	28	41	163	44	48	Slight	Normal	Normal
	405	145	33	41	270	50	55	Normal	Normal	Normal

Appendix V. Individual Microscopic Findings

Animal ID	Heart	Liver	Kidney	Spleen	Brain	Other comments
101	0	0	0	0	0	
102	0	0	0	0	0	
103	0	0	0	0	0	
104	0	0	0	0	0	
105	0	0	0	0	0	
201	1	0	0	0	0	Epicardial chronic inflammation (AV junction)
202	0	0	0	0	1	White matter vacuolation
203	0	0	0	0	1	White matter vacuolation
204	0	0	0	0	1	White matter vacuolation
205	0	0	0	0	1	White matter vacuolation
301	0	0	0	0	0	
302	0	0	0	0	0	
303	0	0	0	0	0	focal mineral in kidney
304	0	0	0	0	0	
305	0	0	0	0	0	
401	0	0	0	0	1	White matter vacuolation
402	0	0	0	0	1	White matter vacuolation
403	0	0	0	0	1	White matter vacuolation
404	0	0	0	0	1	White matter vacuolation
405	0	0	0	0	1	White matter vacuolation

0 = no lesion
1 = mild
2 = moderate
3 = severe

Evaluation of Brain Following a Single Intravenous Dose of UM_COU in Rats

Study Number: 2022109

Michigan State University In Vivo Facility
East Lansing, Michigan 48824

**Sponsor:**	**Peter J.H. Scott, PhD.**<br>**Division of Nuclear Medicine/Department of Radiology**<br>**University of Michigan Medical School**
**Sponsor Contact:**	**Peter J.H. Scott, PhD.**

APPROVAL SIGNATURES

The following individuals confirm that this report accurately represents their interpretation of the data:

Date: 2022.12.21
16:41:00 -05'00'

12/21/2022

Teresa Krieger-Burke, PhD, DVM — Date

TABLE OF CONTENTS

APPROVAL SIGNATURES 2
TABLE OF CONTENTS 3
1. OBJECTIVE 4
2. MATERIALS AND METHODS 4
  2.1. Test Article 4
  2.2. Dose Preparation 4
  2.3. Animal Information 4
  2.4. Experimental Schedule 5
  2.5. Description of Experimental Procedure 5
  2.6. Statistical Analyses 6
3. RESULTS 6
  3.1. Clinical Observations 6
  3.2. Body and Brain Weights 6
  3.3. Brain Histopathology 7
4. DISCUSSION 9
5. CONCLUSION 9

Evaluation of Brain Following a Single Intravenous Dose of UM_COU in Rats

1. OBJECTIVE

The objective of this study was to investigate potential adverse effects on brain histology following a single intravenous dose of UM_COU in male and female rats.

2. MATERIALS AND METHODS

2.1. Test Article

Compound:	UM_COU
Lot Number:	TRK50
Active Moiety (used for dose calculations):	100% assumed
Vehicle:	5% USP Ethanol, 95% 0.9% USP Saline, 10mM NAOAc , pH 5
Dose:	86 µg/kg
Dose Volume:	5 mL/kg
Dose Route of Administration:	Intravenous
Storage Conditions:	Refrigerated, protected from light

2.2. Dose Preparation

Formulations and vehicle were provided by Sponsor ready-to-dose. All formulations were stored under refrigerated conditions, protected from light, until time of use. Formulation were used within 7 days of receipt.

2.3. Animal Information

Eight (8) male and eight (8) female Sprague-Dawley rats were obtained from Charles River Laboratories and weighed 212-225 (males) and 199-215 (females) at the time of dosing initiation. Animals were pair housed in solid bottom cages with aspen bedding for the duration of the study. All animals had free access to standard rodent chow and fresh water throughout the study. Animals were maintained on an automated 12/12 hour light/dark cycle with 7:00am as the start of the light phase.

This study was conducted in accordance with the current guidelines for animal welfare (Guide for the Care and Use of Laboratory Animals, 8th Ed., 2011). The procedures used in this study have been reviewed and approved by the Institutional Animal Care and Use Committee.

2.4. Experimental Schedule

Animals were assigned to an experimental schedule according to Table 1 below.

Table 1. Treatment Schedule

Group	No. of Animals	Animal Numbers	Sex	Treatment	Scheduled Necropsy Day
1	2	101-102	M	vehicle	Day 2
2	2	201-202	F	vehicle	Day 2
3	2	301-302	M	UM_COU 86 µg/kg	Day 2
4	2	401-402	F	UM_COU 86 µg/kg	Day 2
5	2	501-502	M	vehicle	Day 15
6	2	601-602	F	vehicle	Day 15
7	2	701-702	M	UM_COU 86 µg/kg	Day 15
8	2	801-802	F	UM_COU 86 µg/kg	Day 15

Test Subject Arrival Date:	October 11, 2022
In-Life Start Date (Study Day 1):	October 18, 2022
End Date (Completion of In-Life Phase):	November 1, 2022
Data Collection Rooms:	B-23 and B-346, Life Sciences building, MSU

Clinical Observations: pre-dose and daily thereafter
Body Weights: prior to dosing and at time of necropsy
Organ/Tissue Collection: Brain at time of necropsy
Disposition of Animals at Study Completion: Euthanized and carcasses incinerated

2.5. Description of Experimental Procedure

Eight (8) male and eight (8) female Sprague-Dawley rats were used on study. Animals were received from the supplier and were housed in room B-23 in the vivarium of the Life Sciences building at Michigan State University. Animals were pair housed in plastic solid bottom cages containing aspen bedding, with free access to standard rodent chow and fresh water throughout the study. Animals were maintained on an automated 12/12 hour light/dark cycle with 7:00am as the start of the light phase.

Animals acclimated for 1 week. All animals were dosed on October 17, 2022, considered Study Day 1. Animals were lightly anesthetized using isoflurane in oxygen and received a single intravenous bolus in a dose volume of 5 mg/kg of vehicle or UM_COU at 86 µg/kg, according to group assignment. Animals were recovered from anesthesia immediately following dosing.

Clinical observations were recorded daily.

Animals were euthanized by thoracotomy under isoflurane anesthesia on Study Day 2 (Groups 1 through 4) and Study Day 15 (Groups 5 through 8). Terminal body weights were recorded. Brain was collected, weighed, and immediately fixed in 10% neutral-buffered formalin. Brain specimens were delivered on the day of collection to the MSU Investigative Histopathology Laboratory for processing and slide

preparation. Slides were stained with Hematoxylin & Eosin, and reviewed by a board-certified veterinary pathologist.

2.6. Statistical Analyses

Statistical comparisons were not conducted due to the limited number of animals in each treatment group.

3. RESULTS

3.1. Clinical Observations

There were no drug-related clinical findings. All animals remained within normal limits throughout the study.

3.2. Body and Brain Weights

Body weight gain over time in males and females treated with UM_COU was similar to corresponding vehicle controls (Figure 1, Table 2).

Figure 1. Body Weight

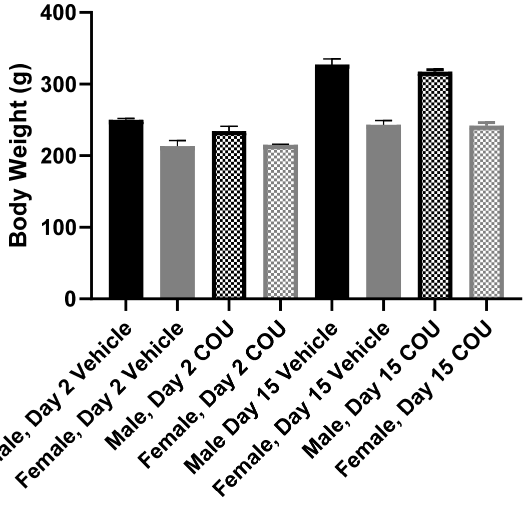

Figure 2. Brain Weight

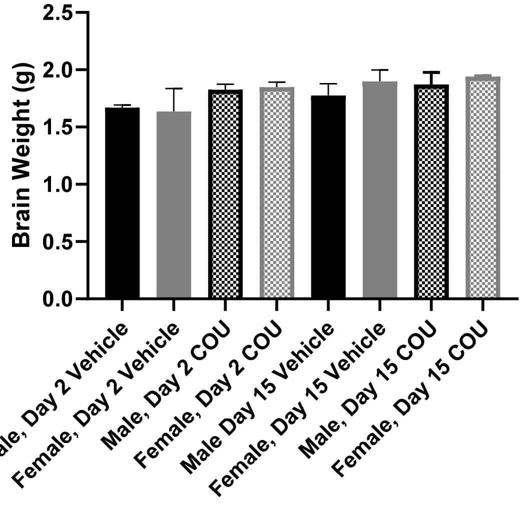

Brain weights and brain-to-body weight ratios of UM_COU-treated males and females were similar to corresponding vehicle controls (Figure 2, Table 2).

Table 2. Brain Weights and Terminal Body Weights

Group	Animal No.	Body Weight (g)	Brain Weight (g)	Brain:Body Weight Ratio
1 Males, Vehicle Day 2	101	252	1.65	0.007
	102	248	1.69	0.007
	Mean	**250**	**1.67**	**0.007**
2 Females, Vehicle Day 2	201	221	1.44	0.006
	202	206	1.84	0.009
	Mean	**214**	**1.64**	**0.008**
3 Males, UM_COU Day 2	301	241	1.87	0.008
	302	228	1.78	0.008
	Mean	**235**	**1.83**	**0.008**
4 Females, UM_COU Day 2	401	216	1.89	0.009
	402	214	1.80	0.008
	Mean	**215**	**1.85**	**0.009**
5 Males, Vehicle Day 15	501	320	1.67	0.005
	502	335	1.88	0.006
	Mean	**328**	**1.78**	**0.005**
6 Females, Vehicle Day 15	601	237	1.80	0.008
	602	249	2.00	0.008
	Mean	**243**	**1.90**	**0.008**
7 Males, UM_COU Day 15	701	315	1.98	0.006
	702	320	1.76	0.006
	Mean	**318**	**1.87**	**0.006**
8 Females, UM_COU Day 15	801	238	1.95	0.008
	802	246	1.93	0.008
	Mean	**242**	**1.94**	**0.008**

3.3. Brain Histopathology

Minimal to mild vacuolation of white matter tracts was noted in all animals. White matter from the diencephalon (corpus collosum and thalamic regions) was particularly affected. The vacuoles varied in size, but were generally small (5-10 µm), crisp, and had a clear center, devoid of debris or membranous material. Rare perineuronal and perivascular regions of vacuolation (edema) were also present across all treatment and control groups. Representative photomicrographs are shown below (Figures 3 and 4).

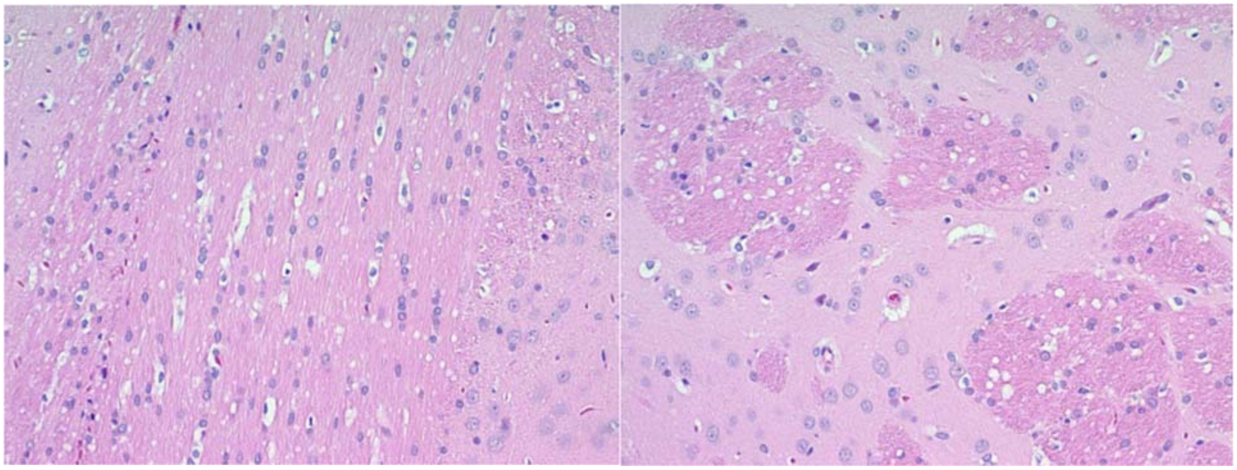
Figure 3. Representative white matter vacuolation in vehicle-treated brain (Rat 102)

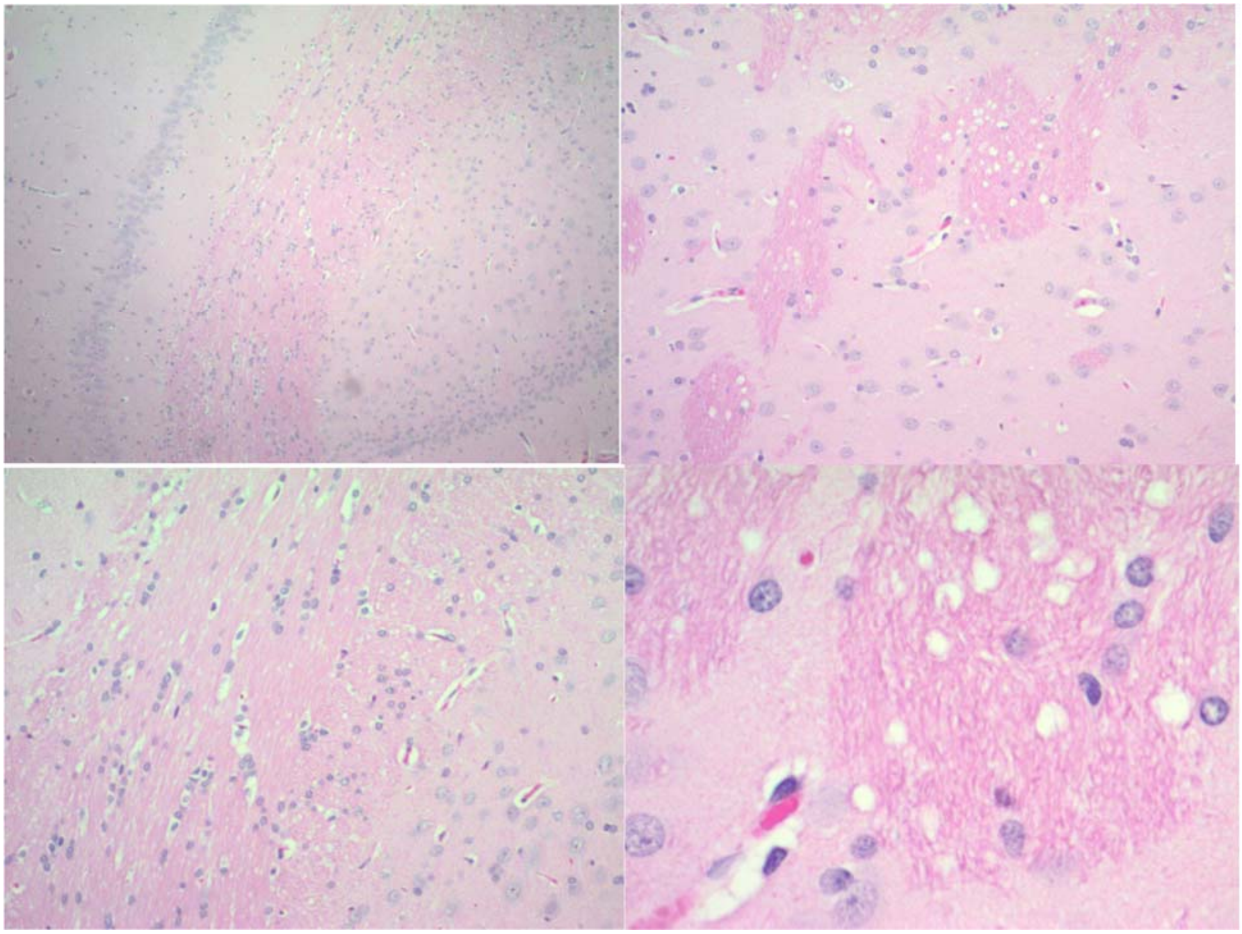
Figure 4. Representative white matter vacuolation in UM_COU-treated brain (Rat 802)

4. DISCUSSION

A screening study of UM_COU in rats (study 2022101) was conducted at the same facility prior to the current study. White matter vacuolation was observed in brain following a single intravenous bolus of UM_COU at 86 µg/kg in the former study. However, only compound-treated rats were evaluated due to the screening nature of the study. The current study investigated whether vacuolation in brain was an adverse effect of the compound by comparing compound-treated brain specimens to vehicle-treated specimens. Because vehicle-treated animals had brain lesions similar to compound-treated, it was determined that these lesions not directly compound related. Instead, the vacuolation in brain appears to be vehicle-related or to be due to specimen processing. Similarities in central nervous system lesions associated with toxicity versus processing and fixation artifact have been described (Kaufmann W, Bolon B, Bradley A, Butt M, Czasch S, Garman RH, George C, Gröters S, Krinke G, Little P, McKay J, Narama I, Rao D, Shibutani M, Sills R. Proliferative and nonproliferative lesions of the rat and mouse central and peripheral nervous systems. Toxicol Pathol. 2012 Jun;40(4 Suppl):87S-157S). Moreover, white matter vacuolation is a very common postmortem artifact associated with fixation, if there is excessive exposure to alcohol (https://ntp.niehs.nih.gov/nnl/nervous/brain/index.htm). The fact that formulated COU and vehicle both contained 5% ethanol USP (v/v), offers a possible explanation.

5. CONCLUSION

UM_COU was clinically well-tolerated when administered as a single intravenous bolus at 86 µg/kg to male and female adult Sprague-Dawley rats, evaluated through 15 days after dosing. There were no adverse effects on body weight gain or on brain weight. Minimal to mild vacuolation of brain white matter was observed in all animals, with no distinction between compound-treated and vehicle-treated animals. Taken together with results from study 2022101, where male and female Sprague-Dawley rats were treated with UM_COU in a similar manner, 86 µg/kg is now considered a no-observed-adverse-effect dose of UM_COU when administered as a single intravenous bolus to rats.
